# Mechanisms of amino acid-mediated lifespan extension in *Caenorhabditis elegans*

**DOI:** 10.1186/s12863-015-0167-2

**Published:** 2015-02-03

**Authors:** Clare Edwards, John Canfield, Neil Copes, Andres Brito, Muhammad Rehan, David Lipps, Jessica Brunquell, Sandy D Westerheide, Patrick C Bradshaw

**Affiliations:** Department of Cell Biology, Microbiology and Molecular Biology, University of South Florida, Tampa, FL 33620 USA

**Keywords:** Amino acids, Lifespan, Aging, *C. elegans*, Serine, Proline, Histidine, Tryptophan, Mitochondrial

## Abstract

**Background:**

Little is known about the role of amino acids in cellular signaling pathways, especially as it pertains to pathways that regulate the rate of aging. However, it has been shown that methionine or tryptophan restriction extends lifespan in higher eukaryotes and increased proline or tryptophan levels increase longevity in *C. elegans*. In addition, leucine strongly activates the TOR signaling pathway, which when inhibited increases lifespan.

**Results:**

Therefore each of the 20 proteogenic amino acids was individually supplemented to *C. elegans* and the effects on lifespan were determined. All amino acids except phenylalanine and aspartate extended lifespan at least to a small extent at one or more of the 3 concentrations tested with serine and proline showing the largest effects. 11 of the amino acids were less potent at higher doses, while 5 even decreased lifespan. Serine, proline, or histidine-mediated lifespan extension was greatly inhibited in *eat-2* worms, a model of dietary restriction, in *daf-16*/FOXO, *sir-2.1*, *rsks-1* (ribosomal S6 kinase), *gcn-2*, and *aak-2* (AMPK) longevity pathway mutants, and in *bec-1* autophagy-defective knockdown worms. 8 of 10 longevity-promoting amino acids tested activated a SKN-1/Nrf2 reporter strain, while serine and histidine were the only amino acids from those to activate a hypoxia-inducible factor (HIF-1) reporter strain. Thermotolerance was increased by proline or tryptophan supplementation, while tryptophan-mediated lifespan extension was independent of DAF-16/FOXO and SKN-1/Nrf2 signaling, but tryptophan and several related pyridine-containing compounds induced the mitochondrial unfolded protein response and an ER stress response. High glucose levels or mutations affecting electron transport chain (ETC) function inhibited amino acid-mediated lifespan extension suggesting that metabolism plays an important role. Providing many other cellular metabolites to *C. elegans* also increased longevity suggesting that anaplerosis of tricarboxylic acid (TCA) cycle substrates likely plays a role in lifespan extension.

**Conclusions:**

Supplementation of *C. elegans* with 18 of the 20 individual amino acids extended lifespan, but lifespan often decreased with increasing concentration suggesting hormesis. Lifespan extension appears to be caused by altered mitochondrial TCA cycle metabolism and respiratory substrate utilization resulting in the activation of the DAF-16/FOXO and SKN-1/Nrf2 stress response pathways.

**Electronic supplementary material:**

The online version of this article (doi:10.1186/s12863-015-0167-2) contains supplementary material, which is available to authorized users.

## Background

In *C. elegans* nematodes free amino acid concentrations change with age [1] and are altered in long-lived worms [2]. In humans, altered plasma amino acid concentrations are biomarkers of several diseases [3] such as type 2 diabetes [4]. Calorie restriction has long been known to delay aging [5] and protein restriction may be responsible for around half of this effect [6]. Methionine [7,8] or tryptophan [9,10] restriction partially mimics protein restriction to extend lifespan and delay aging-related disease in rodents. But the role that other amino acids play in longevity and disease has been harder to elucidate. In this regard, experiments with yeast, worms, and fruit flies are increasingly being used to address this issue.

Using the yeast *Saccharomyces cerevisiae*, it was first discovered that supplementation with the branched chain amino acids (leucine, isoleucine, or valine) or threonine extended chronological lifespan by downregulating the general amino acid control (GAAC) pathway [11]. Others found that glutamate supplementation extended chronological lifespan [12]. Consistent with the ability of glutamate to extend lifespan, deletion of genes involved in converting glutamate to gamma-aminobutyric acid (GABA) increased replicative lifespan [13] and led to increased conversion of glutamate to alpha-ketoglutarate and other TCA cycle intermediates, which may be involved in lifespan extension by maintaining mitochondrial respiratory function. Others using different conditions found that supplementation with serine, threonine, or valine decreased chronological lifespan [14] while limitation of asparagine [15], methionine, aspartate, or glutamate [12] extended lifespan. Further research using yeast deletion strains of differing lifespans found that intracellular levels of many amino acids positively correlated with lifespan [16].

In *Drosophila*, dietary restriction (DR) or protein restriction [17] extends lifespan and supplementing methionine in combination with one or more of the essential amino acids decreased the lifespan back to the fully fed level [18]. Interestingly, adding methionine by itself to DR flies increased protein translation [19] and fecundity [18] without decreasing lifespan, uncoupling these events. Increased levels of amino acids, especially leucine [20,21], activate the TOR kinase, which leads to an increased rate of translation. Inhibition of the TOR kinase with rapamycin [22] or expressing a dominant negative p70-S6 kinase, a kinase downstream of TOR, extended organismal longevity [23]. Metabolism of sulfur containing amino acids was shown to be essential for DR-mediated longevity in *Drosophila* [19], but supplementation of cysteine or methionine failed to extend lifespan in fully fed *Drosophila* [24,25]. However, supplementing casein and methionine together led to lifespan extension [24].

In *C. elegans*, proline supplementation extended lifespan that relied upon its catabolism and a transient increase in reactive oxygen species (ROS) production from the mitochondrial electron transport chain [26]. Increased tryptophan levels also increased longevity in *C. elegans* as knockdown of an enzyme that catabolizes tryptophan increased lifespan [27]. Unexpectedly, knockdown of an aromatic amino acid transporter also extended lifespan [28], suggesting that decreased tryptophan or other aromatic amino acid levels may also boost longevity. Others found that decreased tyrosine degradation led to increased longevity, but surprisingly supplementation of tyrosine to the culture medium did not extend lifespan [29]. The majority of amino acid pool sizes are upregulated in long-lived worms [2]. In *daf-2* insulin-receptor deficient worms, for example, the levels of 8 of the 12 measured amino acids were increased, including the 3 branched chain amino acids. The branched chain amino acids are of special interest for longevity research, since their levels decreased to wild-type levels in the normal-lived *daf-2*/*daf-16* double mutants [2].

Feeding mice a diet high in branched chain amino acids led to increased mitochondrial biogenesis in muscle, decreased ROS production, and increased average lifespan of males [30]. However, branched chain amino acid levels declined in long-lived metformin-treated worms [31], and increased plasma levels of branched chain amino acids are correlated with the development of insulin resistance and type 2 diabetes in humans [32]. Furthermore, studies correlating high levels of free amino acids with longevity must be interpreted with caution as a decreased rate of translation is frequently associated with or even required for longevity and the increased amino acid pools may just be a result of that decreased rate of protein synthesis [33,34].

Due to the incomplete knowledge of the effects of amino acids on longevity as well as the widespread use of amino acid and protein supplementation in the human diet we determined the effects of individual amino acid supplementation on *C. elegans* lifespan. We found that the vast majority of amino acids extended lifespan and further determined many of the signaling pathways required. We then tested the ability of several amino acids or tryptophan catabolites to induce a heat shock response, the ER stress response, or the mitochondrial unfolded protein response, which frequently accompany lifespan extension. The amino acids that extended lifespan to the greatest extent were then tested for effects on stress resistance and proteotoxicity.

## Results

### The effects of individual L-amino acids on the lifespan of *C. elegans*

**Figure 1:**
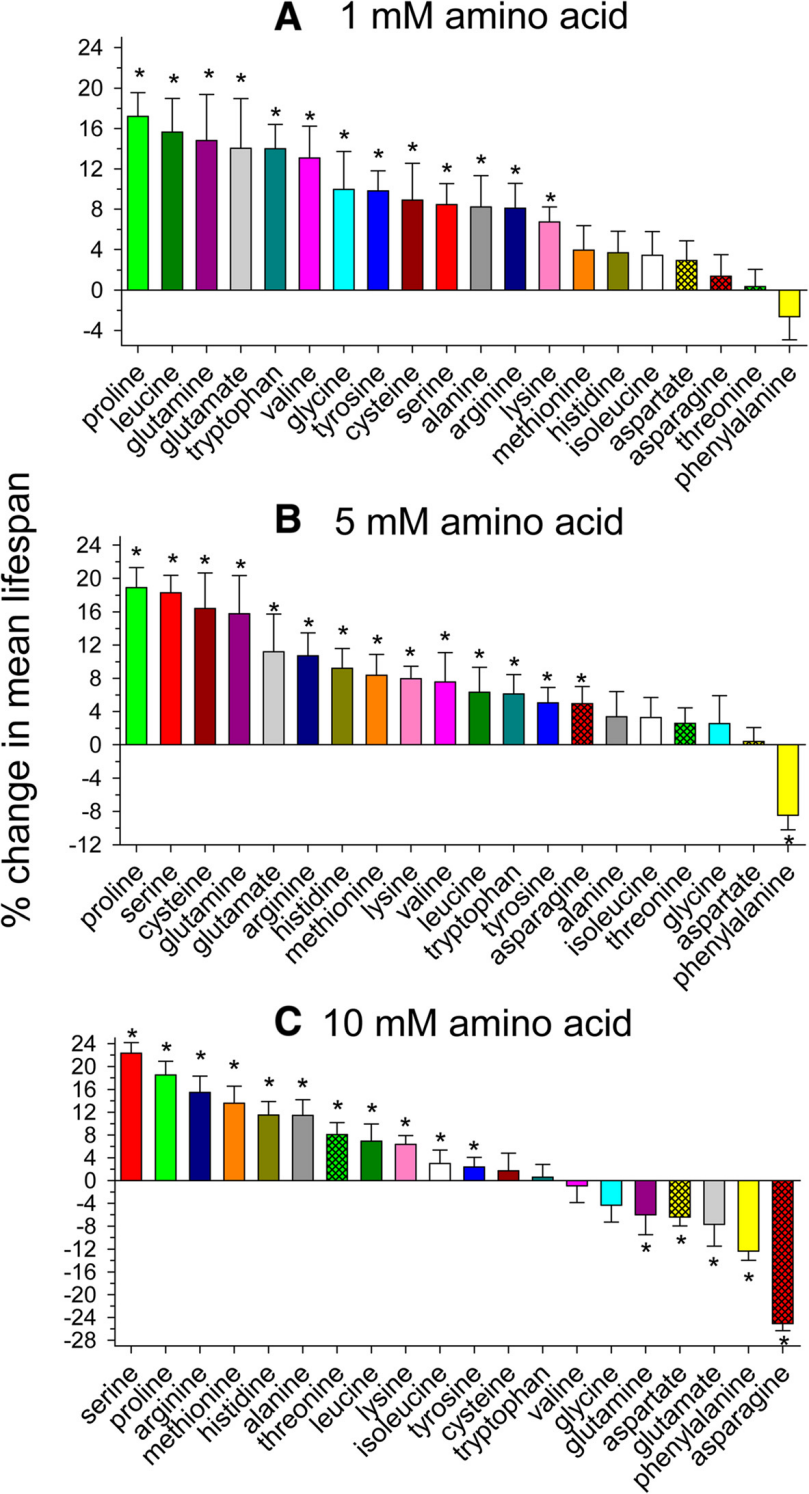
**Individual supplementation of most amino acids extends mean lifespan in** ***C. elegans*** **.** Mean lifespan of *C. elegans* supplemented with a **(A)** 1 mM, **(B)** 5 mM, or **(C)** 10 mM concentration of each of the 20 amino acids (* log rank p < 0.05 vs. control).

We determined the effects of individually supplementing the 19 L-amino acids or glycine on the lifespan of *C. elegans* at 1 mM (Figure 1A), 5 mM (Figure 1B), and 10 mM (Figure 1C) concentrations. The percent change of mean lifespan compared to that of untreated controls performed at the same time is also shown as a table (Additional file 1: Table S1). *C. elegans* worms were grown in liquid S medium with heat-killed *E. coli* as food. Heat killing prevented or at least greatly reduced bacterial catabolism of the added amino acid. Unlike nematode growth medium (NGM) which is standardly used, the S medium contains no peptone, so the bacterial food source and the supplemented amino acid are the only sources of dietary amino acids.

The worms feeding on heat-killed bacteria had a mean lifespan of 17.2 +/− 0.3 days. At a 1 mM concentration, the amino acids that extended lifespan to the greatest extent (14-17%) were proline, leucine, glutamine, glutamate, and tryptophan. At a 5 mM concentration, the greatest lifespan extension was observed with proline, serine, cysteine, and glutamine (16-19%). At this concentration phenylalanine decreased lifespan (8%). Lastly, at a 10 mM concentration, the greatest increases in longevity were observed with serine, proline, arginine, and methionine addition (14-22%). Asparagine, aspartate, phenylalanine, glutamine, and glutamate decreased lifespan at this concentration (6-25%). 5 of the amino acids increased lifespan with increasing concentration from 1 to 10 mM (arginine, histidine, methionine, threonine, and serine), while 7 of the amino acids decreased lifespan with increasing concentration in this range (aspartate, glutamate, glycine, phenylalanine, tryptophan, tyrosine, and valine). 3 of the amino acids had the greatest lifespan extension at the 5 mM concentration (asparagine, cysteine, and glutamine), while the 5 mM concentration yielded the least lifespan extension for alanine. Example lifespan curves for serine, proline, histidine, and tryptophan at concentrations that yielded the greatest effects on mean lifespan are shown (Additional file 2: Figure S1).

### The rate of amino acid uptake may limit the effect on lifespan

To determine if the rate of transport of amino acids into the worms may have limited their effects on lifespan, we administered the cell-permeable histidine analogs N-acetyl-histidine or histidine methylester, which get cleaved by intracellular enzymes to form histidine and monitored lifespan (Table 1). These compounds yielded greater lifespan extension than histidine at the 1 mM dose, suggesting that the rate of transport of the amino acids into the worms is likely limiting their effect on lifespan. The highest concentration of histidine methyl ester (10 mM) did not extend lifespan as expected for a hormetic dose response. If the same observation made for histidine holds for other amino acids, then the rate of amino acid absorption by the intestine may be an important factor controlling their ability to extend lifespan. The rate of transport of hydrophilic antioxidant compounds into *C. elegans* has also been shown to limit their effect on lifespan [35].

**Table 1:** **The effects of D-amino acids and membrane-permeable L-histidine analogs on** ***C. elegans*** **N2 lifespan**

**Treatment**	**Concentration**	**% of untreated mean lifespan**	**p-value**	**# of worms**	**Replicates**
D-alanine	1 mM	114	<0.001	219	2
	5 mM	116	<0.001	273	2
	10 mM	116	<0.001	240	2
D-aspartate	1 mM	107	0.024	233	2
	5 mM	118	<0.001	269	2
	10 mM	108	<0.001	228	2
D-glutamate	1 mM	114	<0.001	207	2
	5 mM	118	<0.001	249	2
	10 mM	97	0.165	232	2
D-serine	1 mM	100	0.600	209	2
	5 mM	91	<0.001	198	2
	10 mM	93	<0.001	220	2
D-proline	5 mM	101	0.734	113	2
N-acetyl-L-histidine	0.1 mM	103	0.383	142	2
	1 mM	112	0.002	188	2
	10 mM	110	0.008	196	2
L-histidine methyl ester	0.1 mM	108	<0.001	215	2
	1 mM	109	<0.001	246	2
	10 mM	104	0.11	193	2

### The effects of D-amino acids on the lifespan of *C. elegans*

To determine if the effects on lifespan were specific for L-amino acids, we also determined if there were effects on longevity when supplementing the 4 D-amino acids found endogenously in *C. elegans* [36], D-alanine, D-serine, D-aspartate, or D-glutamate (Table 1 and Additional file 3: Figure S2) as well as D-proline (Table 1), which is found naturally at low concentrations in mammals [37]. D-alanine and D-asparatate showed greater lifespan extension then their corresponding L-isomers. D-glutamate addition yielded effects on lifespan somewhat similar to its corresponding L-isomer. In contrast to the strong pro-longevity effects observed with L-serine, D-serine supplementation did not lead to lifespan extension at any of the concentrations added and even slightly decreased lifespan at the higher concentrations. And lastly, D-proline supplementation did not extend lifespan as well. These lifespan results are consistent with metabolism of the D-amino acids being required for lifespan extension as D-alanine, D-aspartate, and D-glutamate are present in the *E. coli* food source and have been shown to be catabolized by the products of the *C. elegans* genes *daao-1*, *ddo-1*, and *ddo-3*, respectively. D-serine was not found to be present in the *E. coli* diet, but was instead found to be synthesized endogenously by *C. elegans*. However, no *C. elegans* enzyme has yet been found to mediate D-serine [36] or D-proline degradation.

### Amino acid-mediated lifespan extension, except when induced by tryptophan, is DAF-16 dependent

To determine if lifespan extension induced by amino acids requires specific longevity pathways, individual amino acids were administered to mutants of known longevity pathways at the concentration that maximally extended lifespan. First, the amino acids alanine, cysteine, glutamine, histidine, lysine, proline, serine, tryptophan, and tyrosine were individually supplemented to short-lived *daf-16(mgDf50)* mutants (Table 2). DAF-16/FOXO is a central transcription factor that translocates to the nucleus to activate a stress response program in insulin-receptor signaling-deficient worms [38]. Tryptophan was the only supplemented amino acid that extended lifespan in the *daf-16* mutant strain indicating tryptophan activates a longevity pathway independent of *daf-16*, while the other 8 amino acids require DAF-16 mediated gene expression for the increased longevity. Cysteine and histidine even decreased lifespan when supplemented to this strain.

**Table 2:** **The effects of amino acids on lifespan in** ***daf-16*** **mutant and** ***skn-1*** **knockdown** ***C. elegans***

**Strain**	**Treatment**	**% of N2 mean lifespan**	**% of untreated mean lifespan**	**p-value**	**# of worms**	**Replicates**
*daf-16(mgDf50)*	Control	73		<0.001	608	7
	5 mM histidine		92	<0.001	286	2
	5 mM proline		106	0.0871	133	2
	1 mM alanine		102	0.111	107	2
	1 mM tryptophan		118	0.008	183	2
	10 mM serine		105	0.104	161	2
	10 mM glutamine		95	0.107	125	2
	5 mM cysteine		95	0.107	115	2
	10 mM cysteine		81	<0.001	105	2
	5 mM tyrosine		96	0.578	140	2
	5 mM lysine		99	0.907	130	2
N2 (*skn-1* RNAi) with live bacteria	Control	59		<0.001	156	3
	1 mM tryptophan		107	0.042	125	3
	10 mM serine		107	0.01	175	3
	5 mM histidine		103	0.296	139	3
	5 mM proline		105	0.088	111	2
N2 with live bacteria	Control	-			298	5
	1 mM tryptophan		109	<0.001	317	5
	10 mM serine		109	<0.001	375	5
	5 mM histidine		110	<0.001	277	5
	5 mM proline		108	<0.001	268	4

**Figure 2:**
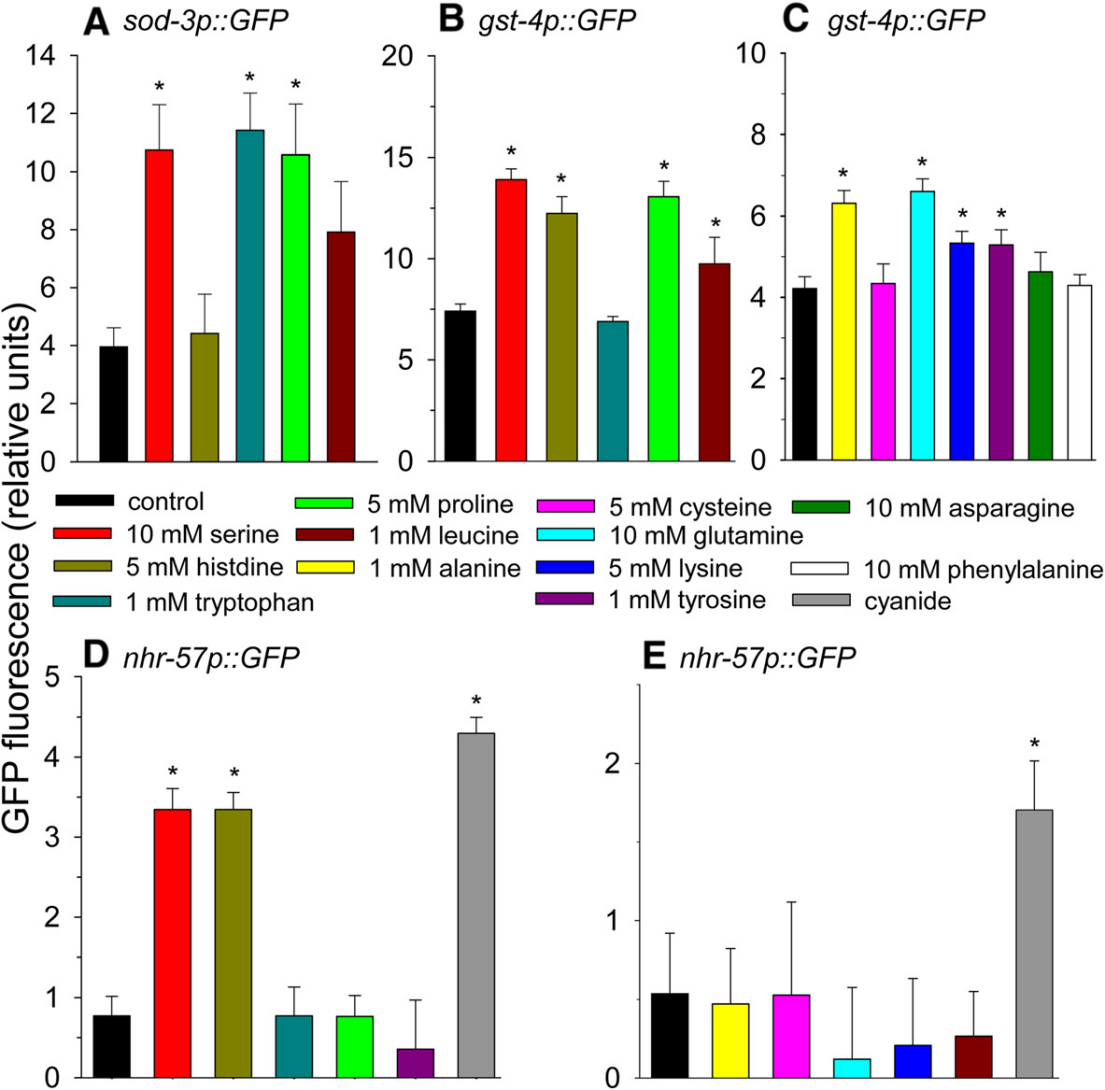
**The effects of amino acid addition on DAF-16, SKN-1, and HIF-1-mediated gene expression. (A)** The effects of amino acid addition on *sod-3p::GFP* fluorescence as a measure of DAF-16 transcriptional activity. **(B)** and **(C)** The effects of amino acid addition on *gst-4p::GFP* fluorescence as a measure of SKN-1 transcriptional activity. **(D)** and **(E)** The effects of amino acid addition on *nhr-57p::GFP* fluorescence as a measure of HIF-1 transcriptional activity. 20 μM potassium cyanide was used as a positive control (*p < 0.05).

To confirm that amino acids activate DAF-16 transcriptional activity, we measured the fluorescence of a *sod-3p::gfp* DAF-16 reporter strain of worms following culture in the presence of individual amino acids (Figure 2A). As expected from the lifespan data, serine and proline increased fluorescence of these worms. The presence of tryptophan also increased fluorescence, so tryptophan likely activates both DAF-16-dependent and DAF-16-independent pathways for lifespan extension. There was also a strong trend for leucine to increase fluorescence. Unexpectedly, histidine did not increase expression of *sod-3p::gfp*. The reasons for this finding are unclear as we found that DAF-16 was required for histidine-mediated lifespan extension.

### Most amino acids activate SKN-1 transcriptional activity

The SKN-1/Nrf2 signaling pathway increases cellular antioxidant and detoxification gene expression to extend lifespan. We determined the effect of serine, tryptophan, histidine, or proline addition on lifespan of *skn-1* knockdown worms. As shown in Table 2, tryptophan or serine supplementation extended lifespan, but not to the extent as in control worms, while there were only insignificant trends toward increased lifespan following histidine or proline addition.

**Table 3:** **The effects of amino acids on lifespan in aak-2,** ***sir-2.1*** **and** ***eat-2*** **mutant and** ***bec-1*** **knockdown worms**

**Strain**	**Treatment**	**% of N2 control mean lifespan**	**% of untreated mean lifespan**	**p-value**	**# of worms**	**Replicates**
*aak-2(gt33)*	Control	80		<0.001	786	8
	5 mM histidine		100	0.877	189	2
	5 mM proline		100	0.919	187	2
	1 mM alanine		100	0.943	209	2
	1 mM tryptophan		102	0.060	220	2
	10 mM serine		101	0.137	204	2
	5 mM lysine		87	0.373	180	2
	5 mM cysteine		85	0.952	140	2
	10 mM cysteine		90	0.122	145	2
	5 mM tyrosine		80	<0.001	200	2
	5 mM glutamine		94	0.436	180	2
	10 mM glutamine		103	0.126	200	2
*sir-2.1(ok434)*	Control	86		<0.001	299	4
	5 mM histidine		100	<0.830	154	2
	5 mM proline		104	0.211	114	2
	1 mM alanine		98	0.225	165	2
	1 mM tryptophan		102	0.673	121	2
	10 mM serine		101	0.482	136	2
	10 mM glutamine		110	<0.001	180	2
	5 mM cysteine		105	0.244	192	2
	10 mM cysteine		110	<0.001	190	2
	5 mM tyrosine		93	0.231	131	2
	5 mM lysine		107	0.017	146	2
	1 mM phenylalanine		105	0.049	155	2
	10 mM phenylalanine		101	0.402	176	2
*eat-2(ad1116)*	Control	142		<0.001	135	2
	10 mM serine		108	0.078	161	2
	1 mM tryptophan		105	0.099	138	2
	5 mM glutamine		100	0.841	134	2
	5 mM histidine		106	0.091	153	2
	5 mM proline		107	0.076	176	2
N2 (*bec-1* RNAi) with live bacteria	Control	123		<0.001	184	2
	1 mM tryptophan		107	<0.001	162	2
	10 mM serine		101	0.316	152	2
	5 mM histidine		99	0.274	117	2
	5 mM proline		98	0.09	102	2

To further check the ability of amino acids to activate SKN-1, we used a *gst-4p::GFP* SKN-1 reporter strain of worms (Figure 2B and C). We found that 8 of the 10 amino acids tested that increased lifespan increased GFP expression of the reporter strain. Amino acids that activated *gst-4p::GFP* expression included serine, proline, glutamine, alanine, leucine, lysine, and tyrosine, while tryptophan and cysteine did not. We also tested the effects of 2 amino acids, phenylalanine and asparagine, which decreased lifespan on the fluorescence of this reporter strain and observed no induction of expression. Overall, there was a small correlation between the amount of SKN-1 activity and the extent of lifespan extension as serine and proline extended lifespan to the greatest extent and also increased fluorescence of the SKN-1 reporter strain to the greatest extent. It has previously been hypothesized that proline catabolism transiently increases ROS production that leads to SKN-1 activation [26]. Our results are consistent with this hypothesis. Cysteine is a strong antioxidant and likely quenched ROS required for SKN-1 activation likely explaining the lack of activation by this amino acid.

The RNAi feeding experiments require live bacteria, while heat-killed bacteria were used in all other lifespan experiments. It is possible that the live bacteria used in the SKN-1 RNAi lifespan experiments metabolized the added amino acids dampening the degree of lifespan extension. Therefore, we performed control experiments supplementing amino acids to *C. elegans* feeding on live control HT115(DE3) *E. coli. C. elegans* fed live control bacteria had a mean lifespan of 16.1 +/− 0.2 days, slightly less than worms fed heat-killed bacteria (mean lifespan of 17.2 +/− 0.3 days). Histidine extended lifespan to a similar extent in the presence of live or heat-killed bacteria as shown (Table 2 and Additional file 1: Table S1). However, tryptophan-induced lifespan extension was slightly blunted by the use of live bacteria, and serine or proline-induced lifespan extension was blunted by roughly 50% by the use of live bacteria. A faster rate of *E. coli* catabolism of serine and proline than tryptophan and histidine likely explain these observations.

### Histidine and serine increase HIF-1 target gene expression

The hypoxia-inducible factor-1 (HIF-1) protein is degraded quickly during standard conditions, but is stabilized during hypoxia or by other specific stresses to increase lifespan in *C. elegans* [39]. Therefore, we tested the HIF-1 reporter strain *nhr-57p::GFP* [39] for amino acid-induced changes in GFP fluorescence (Figure 2D and E). Cyanide was used as a positive control as it inhibits cytochrome c oxidase, the protein complex which binds molecular oxygen, the terminal electron acceptor in the electron transport chain, to mimic the effects of hypoxia on mitochondria. We found that histidine or serine increased fluorescence, while tryptophan, proline, tyrosine, alanine, cysteine, glutamine, lysine, or leucine did not. These data indicate that stabilization of HIF-1 may be one of the mechanisms through which histidine and serine extend lifespan, although lifespan experiments with HIF-1 mutant worms are needed to confirm this hypothesis.

### Amino acid-mediated lifespan extension is AAK-2 (AMPK) dependent

Next we individually administered *C. elegans* our test set of 10 amino acids except leucine to *aak-2(gt33)* worms, which are depleted of one of the two catalytic subunits of AMP-activated protein kinase (AMPK) and performed lifespan analysis (Table 3). AMPK signaling inhibits target of rapamycin (TOR) kinase signaling to stimulate autophagy to recycle cellular components. AMPK also stimulates the sirtuin deacetylase SIR-2.1, SKN-1/Nrf2, and DAF-16/FOXO pro-longevity pathways [40]. None of the amino acids extended lifespan in this mutant strain. Tyrosine decreased lifespan while the other 8 amino acids tested had no significant effect. Therefore, AAK-2 is required for the longevity benefits provided by the amino acids.

**Table 4:** **The effects of amino acids on lifespan of** ***rsks-1*** **,** ***gcn-2*** **,** ***ife-2*** **,** ***gas-1*** **, and** ***mev-1*** **mutants**

**Strain**	**Treatment**	**% of N2 control mean lifespan**	**% of untreated mean lifespan**	**p-value**	**# of worms**	**Replicates**
*rsks-1(ok1255)*	Control	109		<0.001	362	4
	1 mM tryptophan		71	<0.001	130	2
	5 mM histidine		93	0.031	135	2
	5 mM proline		94	0.042	140	2
	10 mM serine		101	0.236	162	2
*gcn-2(ok871)*	Control	90		<0.001	479	4
	1 mM tryptophan		99	0.489	160	2
	5 mM histidine		100	0.335	163	2
	5 mM proline		107	0.001	205	2
	10 mM serine		104	0.006	183	2
*Ife-2(ok306)*	Control	97		0.245	112	2
	1 mM tryptophan		94	0.029	138	2
	5 mM histidine		98	0.597	110	2
	5 mM proline		102	0.352	117	2
	10 mM serine		99	0.791	96	2
*gas-1(fc21)*	Control	69		<0.001	119	2
	5 mM histidine		104	0.092	110	2
	10 mM serine		98	0.327	106	2
	5 mM proline		109	0.003	115	2
*mev-1(kn1)*	Control	69		<0.001	273	2
	5 mM histidine		104	0.021	215	2
	10 mM serine		109	<0.001	258	2
	5 mM proline		102	0.102	256	2

### Many amino acids require SIR-2.1 for lifespan extension

Next the effects of individual supplementation of these same 9 amino acids as well as phenylalanine on lifespan of the *sir-2.1(ok434)* NAD-dependent sirtuin deacetylase mutant were determined (Table 3). Small to moderate lifespan increases occurred with cysteine, glutamine, lysine, and low dose phenylalanine supplementation, but there were no significant effects of 6 other amino acids tested on the lifespan of this strain. Therefore SIR-2.1 was required for lifespan extension mediated by slightly more than half of the amino acids tested. Surprisingly, high dose (10 mM) phenylalanine supplementation did not lead to a decreased lifespan in this strain as it did in the N2 control worms.

### Amino acids do not significantly extend lifespan in long-lived DR worms

Restricting a specific amino acid such as methionine from the diet can be utilized to extend lifespan and gain some of the benefits of dietary restriction (DR) [8], but there is not much evidence that specific amino acid supplementation can yield enhanced longevity effects. Therefore, we administered individual amino acids to *eat-2(ad1116)* mutants that are dietarily restricted and long-lived because of reduced pharyngeal pumping. The non-treated control *eat-2* mutants had a mean lifespan 42% longer than N2 controls indicating that our control worms were not dietarily restricted under our growth conditions. None of the 5 amino acids tested yielded statistically significant lifespan extension (Table 3). However, 4 of the amino acids yielded strong trends toward lifespan extension (p-values between 0.08 and 0.10). Therefore the individual amino acids are likely utilizing some portion of the DR signaling pathway for lifespan extension.

### Autophagy is required for the lifespan extension induced by serine, proline, or histidine supplementation, but not by tryptophan

Since autophagy has been shown to be required for DR-mediated lifespan extension [41], we determined if autophagy was also required for amino acid-mediated longevity. To block autophagy we knocked down *bec-1*, the *C. elegans* Beclin-1 homolog and monitored lifespan following supplementation with serine, proline, histidine, or tryptophan. Knockdown of *bec-1* by RNAi feeding increased lifespan as has previously been shown in [42] and prevented lifespan extension induced by supplementation with serine, proline or histidine, but not by tryptophan (Table 3). Therefore, the majority of amino acids, but not tryptophan, require autophagy for lifespan extension, once again suggesting that tryptophan extends lifespan through a mechanism distinct from other amino acids.

The PHA-4/FOXA transcription factor is required for induction of autophagy and lifespan extension in response to DR. In addition, expression of the PHA-4 transcription factor has been shown to be upregulated by roughly 50% by DR [43]. Therefore, we determined if individual amino acid administration could increase PHA-4 protein levels by using a strain of worms engineered to express PHA-4:GFP:3xFLAG using the endogenous *pha-4* promoter [44]. Surprisingly, we found that serine, histidine, or tryptophan addition did not alter the GFP fluorescence of this strain (Additional file 4: Figure S3). However, leucine addition resulted in a strong trend toward increased fluorescence (p = 0.08). On the whole, individual amino acid supplementation did not appear to have much of an effect on fluorescence in this strain. However, we cannot yet rule out the possibility that changes in PHA-4 localization or post-translational modification play a role in individual amino acid-induced longevity. It is also possible that the added GFP and FLAG tags affect the stability of the protein. Lifespan studies using *pha-4* RNAi are needed to determine a role, if any, for PHA-4 in amino acid-mediated lifespan extension.

### Inhibition of TOR signaling plays a role in amino acid-mediated lifespan extension

Specific amino acids, most notably leucine, but also to a lesser extent arginine and glutamine can be activators of the TOR signaling pathway that limits lifespan [45]. Administering rapamycin, a TOR inhibitor, or feeding TOR RNAi to *C. elegans* induces autophagy and extends lifespan [46,47]. More recently it was found that alpha-ketoglutarate supplementation can lead to TOR inhibition to extend lifespan [48]. Knockout or knockdown of the ribosomal S6 kinase, which is downstream of TOR kinase in the signaling pathway, also extends lifespan [49]. Part of this effect may rely on a decreased rate of protein translation as inhibitors of protein translation can also extend lifespan [49]. Therefore, we determined the effects of specific amino acids on lifespan in long-lived *rsks-1(ok1255)* ribosomal S6 kinase mutants, where this arm of the TOR signaling pathway is inhibited (Table 4). Unlike the results with N2 control worms, addition of serine did not alter the lifespan of this strain, while proline or histidine addition slightly decreased lifespan, and tryptophan addition decreased lifespan by 20%. Therefore, these amino acids appear to use inhibition of TOR signaling to mediate lifespan extension, as the amino acids did not extend lifespan in long-lived mutant worms where TOR signaling was already disrupted.

### A decreased rate of translation is required for the full lifespan extending effects of amino acids

GCN-2 (general control nonderepressible-2) kinase can slow the rate of translation initiation by phosphorylating eukaryotic translation initiation factor-2 alpha (eIF-2α) when tRNAs become uncharged due to low amino acid levels [34] or in times of mitochondrial metabolic stress [33]. We hypothesized that amino-acid supplementation causing amino acid imbalance could result in inefficient tRNA charging or cause metabolic stress signaling through GCN-2 to extend lifespan. Therefore, we determined the lifespan of *gcn-2(ok871)* mutants supplemented with individual amino acids (Table 4). We found that histidine or tryptophan supplementation did not lead to increased longevity when using this strain, while only a 4% or 7% lifespan extension occurred when serine or proline, respectively, were supplemented. To further determine a role for decreased translation in amino acid imbalance-mediated longevity, we performed lifespan analysis using the *ife-2(ok306)* strain [50], which is deficient in an isoform of the translation initiation factor eIF4E and shown to be long-lived. Surprisingly, under our liquid culture conditions using heat-killed bacteria as food, the lifespan of this strain was not significantly different than the control. However, supplementation of serine, proline, histidine, or tryptophan to this strain did not lead to extended lifespan, while tryptophan addition even slightly decreased lifespan. Therefore signaling to slow the rate of translation is likely a general mechanism involved in individual amino acid-mediated increased longevity.

### The effect of individual amino acids on the lifespan of mitochondrial ETC complex I and II mutants

To test the hypothesis that mitochondrial ETC activity is important for amino-acid induced lifespan extension we supplemented serine, histidine, or proline to either short-lived mitochondrial ETC complex I defective *gas-1(fc21)* mutant worms or to short-lived mitochondrial ETC complex II defective *mev-1(kn1)* mutant worms (Table 4). We found that proline supplementation extended the lifespan of *gas-1* mutants, but that serine or histidine were unable to extend lifespan, although there was a strong trend with histidine (p = 0.09). When we supplemented each of these 3 amino acids to *mev-1* mutants, we found opposite effects. Proline did not extend lifespan, although a strong trend was observed (p = 0.10), while serine and histidine extended lifespan. Therefore normal complex I (NADH dehydrogenase) activity is required for the full serine and histidine-mediated lifespan extension, while normal complex II activity is required for proline-mediated lifespan extension. This data may be explained in that proline dehydrogenase generates FADH_2_ which feeds electrons into the ETC at complex II and histidine and serine catabolism generates NADH that feeds electrons into the ETC at complex I.

### Most supplemented metabolites extended lifespan at an optimal concentration

**Figure 3:**
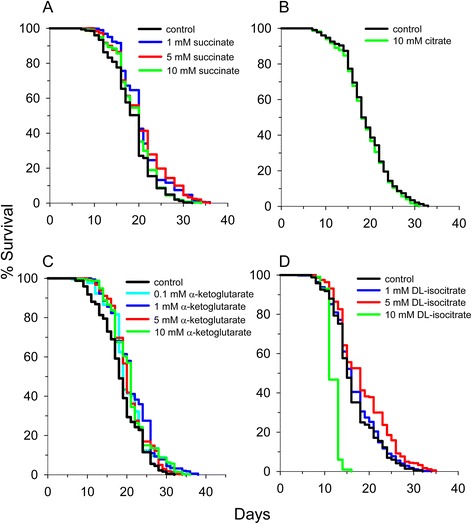
**Individual supplementation of many TCA cycle metabolites extends mean lifespan in** ***C. elegans*** **. (A)** Succinate extends lifespan, **(B)** citrate does not extend lifespan, **(C)** alpha-ketoglutarate extends lifespan, and **(D)** isocitrate extends lifespan at one or more of the concentrations tested. 10 mM citrate is a standard component of the S-medium. It was removed to determine the effect of citrate on lifespan.

Since supplementation with an optimal concentration of most amino acids extended the lifespan of the worms, it is possible that their breakdown to TCA cycle intermediates may play a role in lifespan extension. It has previously been shown that supplementation with pyruvate [51], acetate (that can be readily metabolized to the TCA cycle substrate acetyl-CoA) [52], or the TCA cycle intermediates malate, fumarate [53], oxaloacetate [54], and alpha-ketoglutarate [48] extended lifespan in *C. elegans*. We therefore determined the lifespan of the worms individually supplemented with 1, 5, or 10 mM concentrations of the TCA cycle intermediates citrate, isocitrate, alpha-ketoglutarate, or succinate (Table 5). We previously found that 10 mM succinate did not extend lifespan, but did induce translocation of the pro-longevity factor DAF-16 to the nucleus [53]. But here we find that lowering the concentration of succinate to 5 mM or 1 mM resulted in lifespan extension (Figure 3A), consistent with a recent report of a longevity benefit [48]. Citrate is present at 10 mM in all of our experiments as a standard buffer component of the S-medium. We found that removing it did not affect the lifespan (Figure 3B). Previous findings also failed to find an extension of lifespan with citrate supplementation [48,52]. We found that alpha-ketoglutarate at any of the 4 concentrations tested from 0.1 to 10 mM extended lifespan (Figure 3C), as recently reported for an 8 mM dose [48]. Adding DL-isocitrate to the medium led to an increase in lifespan at the 5 mM concentration, but a decrease in lifespan at the 10 mM concentration (Figure 3D). It is unknown if the non-naturally occuring L-isomer contributed to this effect, but since we found the non-naturally occurring isomer D-malate to decrease lifespan at all 3 concentrations tested (Table 5), it is a strong possibility. Another group observed no effect of 8 mM isocitrate on lifespan [48]. Therefore, of the 7 TCA cycle intermediates that we have tested, 6 were able to extend lifespan at an optimal dose.

**Table 5:** **The effects of TCA cycle intermediates and their isomers on** ***C. elegans*** **lifespan**

**Treatment**	**Concentration**	**% of untreated mean lifespan**	**p-value**	**# of worms**	**Replicates**
succinate	1 mM	111	<0.001	590	4
	5 mM	110	<0.001	570	4
	10 mM	104	0.098	607	4
citrate^1^	10 mM	98	0.414	229	2
α-ketoglutarate	0.1 mM	110	<0.001	261	3
	1 mM	115	<0.001	345	3
	5 mM	111	<0.001	337	3
	10 mM	107	0.042	333	3
DL-isocitrate	1 mM	103	0.202	452	4
	5 mM	113	<0.001	523	4
	10 mM	72	<0.001	217	4
D-malate	1 mM	85	<0.001	106	1
	5 mM	76	<0.001	77	1
	10 mM	79	<0.001	142	1

^1^compared to a medium lacking citrate. All other experiments contain 10 mM citrate as part of the standard culture media.

We hypothesized that catabolism of the amino acids for anaplerosis or energy production was likely playing a role in the lifespan extension. If this is true supplementing other common cellular metabolites should also extend lifespan. Therefore, we performed lifespan analysis of worms supplemented with sugars or other metabolites lacking nitrogen atoms (Additional file 5: Table S2). At an optimal dose gluconate, glycerol, inositol, phophoenolpyruvate, or ribose substantially increased lifespan, while xylose, galactose, DL-lactate, or caprylate just slightly increased lifespan. Glucuronolactone, glyceraldehyde, fructose, propionate, or dihydroxyacetone did not extend lifespan and the last 4 of these compounds even decreased lifespan by 13-25% at the 10 mM dose. Glyceraldehyde, fructose, and dihydroxyacetone are readily converted into glycolytic intermediates leading to the formation of toxic methylglyoxyl from glyceraldehyde phosphate or dihydroxyacetone phosphate, which could contribute to their toxicity, while propionic acid is known to be neurotoxic at high levels [28].

Since many amino acids activated SKN-1, while TCA cycle intermediates did not, we hypothesized that nitrogen-containing metabolites might be slightly more potent inducers of lifespan extension. The effects of many nitrogen-containing metabolites on lifespan are shown in Additional file 6: Table S3. At an optimal dose carnosine, beta-alanine, betaine, homocysteine, ornithine, agmatine, putrescine, taurine, and theanine extended lifespan. For the majority of these compounds, the lowest concentration, such as 1 mM, yielded greater lifespan extension than the highest 10 mM concentration suggesting a hormetic dose response. Supplementation with creatine, or the histidine catabolites histamine or urocanic acid did not extend lifespan at any of the 3 concentrations tested. Although most of the nitrogen-containing compounds extended lifespan at an optimal dose, the extent of lifespan extension was not noticeably different than when supplementing with compounds lacking nitrogen.

### *C. elegans* lifespan was not limited by nitrogen availability

We used heat-killed *E. coli* as a food source to prevent the bacteria from metabolizing the added metabolites, but we have found that heat-killing *E. coli* causes the loss of one or more essential growth-limiting nutrients during heating. So lowering the concentration of heat-killed bacteria in the growth media by just a factor of 2 did not allow completion of larval growth into adulthood, but instead led to dauer formation. The concentration of live bacteria could be reduced by 30–40 fold before dauer formation during larval development. To test if the worms may have been nitrogen limited under our culture conditions, we supplemented the worms with peptone or other nitrogen containing compounds and measured the lifespan. Interestingly, peptone at 1.25 g/L, half the concentration present in nematode growth media (NGM) decreased lifespan by 22% (Additional file 6: Table S3). This concentration contains roughly 10 mM total amino acids. These results support published findings where 5 g/L (2x NGM) and 10 g/L (4x NGM) peptone also decreased *C. elegans* lifespan in liquid S medium [55]. Lowering the peptone concentration to 0.125 g/L (0.1x NGM) yielded a similar lifespan as untreated controls. We next added ammonium chloride as a nitrogen source. Concentrations of ammonium chloride from 1 to 10 mM did not extend lifespan. So amino acids do not increase lifespan solely by providing nitrogen to the worms.

### Phenylalanine and alpha-ketoglutarate activate a HSF-1 reporter strain

**Figure 4:**
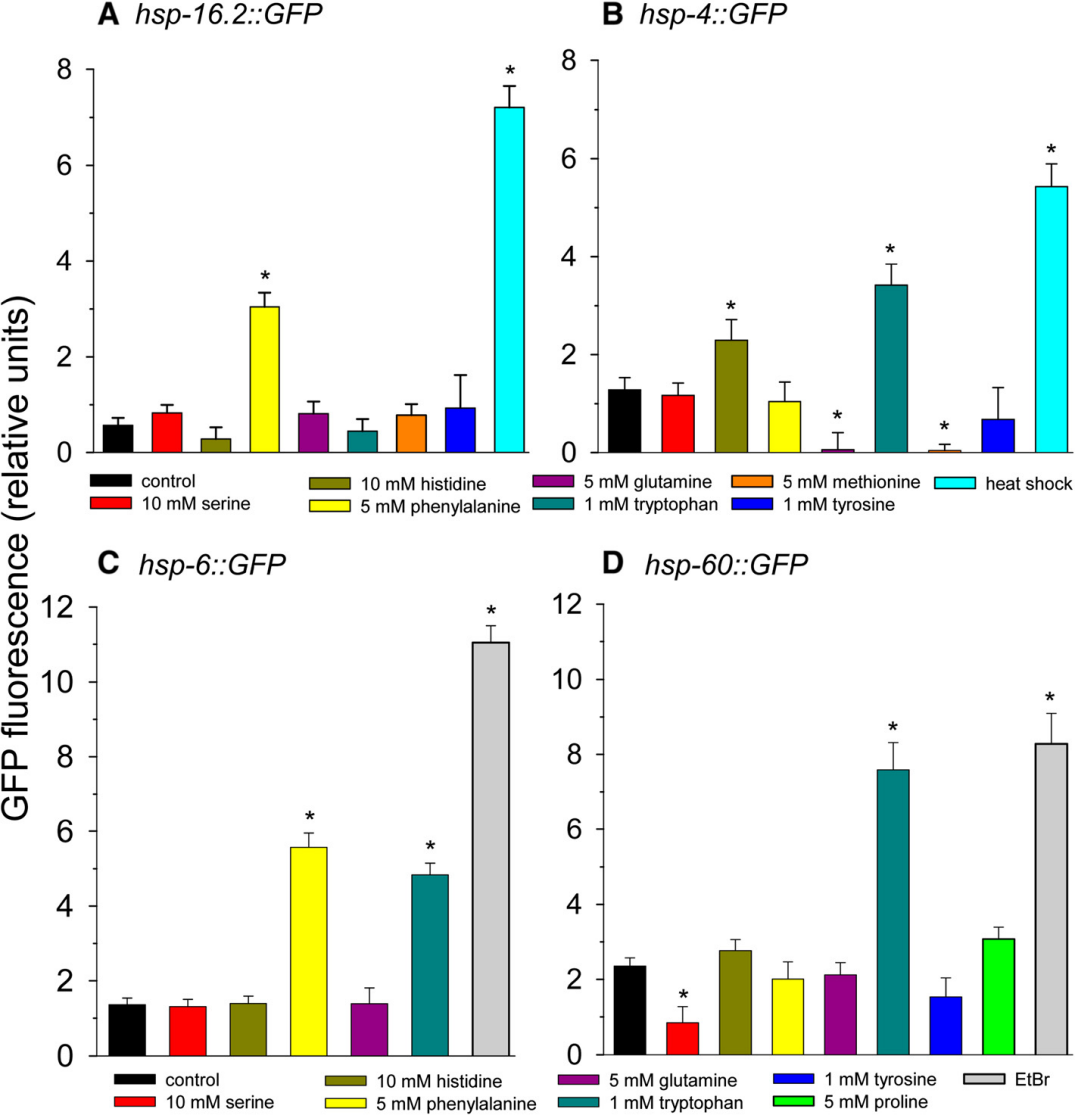
**The effects of amino acids on heat shock, mitochondrial unfolded protein response, and ER stress response.** Amino acids were added at the concentration tested that yielded maximal lifespan extension except phenylalanine, which did not extend lifespan. **(A)** *Phsp-16.2::GFP* **(B)** *Phsp-4::GFP* **(C)** *Phsp-6::GFP* **(D)** *Phsp-60::GFP*. For panels A and B heat shock at 35°C for 2 hours was used as a positive control. For panels C and D 50 μg/ml ethidium bromide treatment for 2 days was used as a positive control (*p < 0.05 vs. control).

Since supplementation of many of the amino acids and other metabolites showed less lifespan extension at higher concentrations, we hypothesized that *C. elegans* mounted a stress response that resulted in lifespan extension at lower amino acid levels, but at higher levels the stress response was overwhelmed leading to decreased lifespan. Therefore, we determined if amino acid supplementation activates a *Phsp-16.2::GFP* heat shock reporter strain of worms. HSP-16.2 is a small cytoplasmic heat shock protein and target of the HSF-1 transcription factor [56]. We first tested the effects of glutamine, histidine, methionine, serine, tryptophan, or tyrosine, amino acids that extended lifespan, or phenylalanine, an amino acid that decreased lifespan on GFP fluorescence in the *Phsp-16.2::GFP* reporter strain using heat shock as a positive control (Figure 4A). Of these amino acids, only phenylalanine activated GFP reporter gene expression.

**Figure 5:**
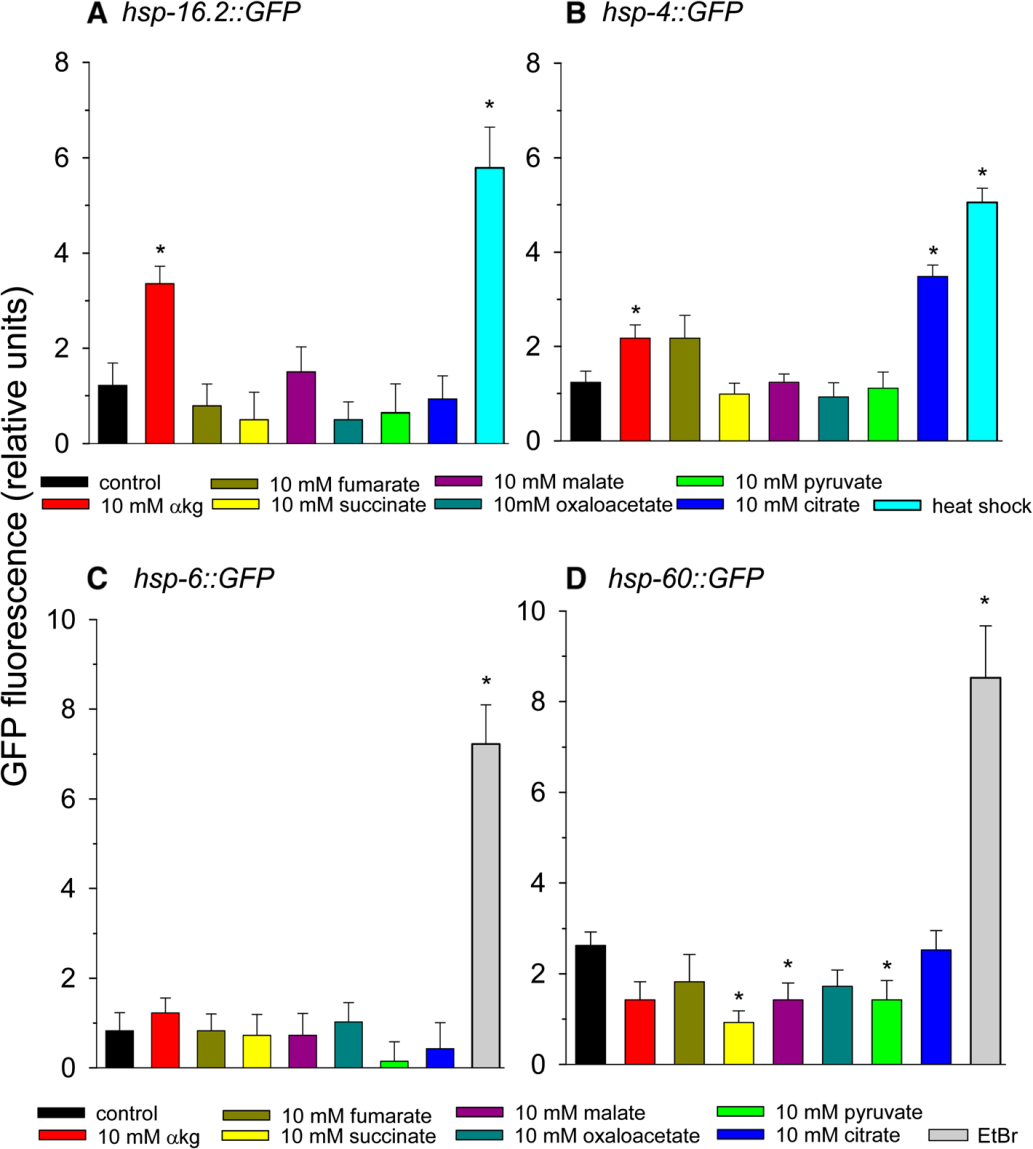
**The effects of TCA cycle metabolites and pyruvate on heat shock, mitochondrial unfolded protein response, and ER stress response. (A)** *Phsp-16.2::GFP* **(B)** *Phsp-4::GFP* **(C)** *Phsp-6::GFP* **(D)** *Phsp-60::GFP* (*p < 0.05 vs. control). For panels A and B heat shock at 35°C for 2 hours was used as a positive control. For panels C and D 50 μg/ml ethidium bromide treatment for 2 days was used as a positive control (*p < 0.05 vs. control).

We next tested the effects of TCA cycle intermediate or pyruvate supplementation on the *Phsp-16.2::GFP* reporter strain, as amino acids are broken down into TCA cycle intermediates when they are present in excess. When administered to the *Phsp-16.2::GFP* reporter strain alpha-ketoglutarate, but none of the other TCA cycle intermediates supplemented increased GFP fluorescence (Figure 5A). Many of the amino acids showing the largest stimulatory effects on lifespan (proline, arginine, histidine, glutamine, and glutamate) are catabolized through glutamate into alpha-ketoglutarate in the TCA cycle.

### Histidine, tryptophan, and citrate induce an ER stress response

We next determined if amino acids activated the endoplasmic reticulum (ER) stress response by using a reporter strain of worms engineered to contain a heat shock protein-4 (*hsp-4*) promoter driving expression of green fluorescent protein (GFP) [57]. We tested the effect of glutamine, histidine, methionine, serine, tryptophan, or tyrosine supplementation on expression of GFP in the *Phsp-4::GFP* reporter strain of worms using heat shock as a positive control (Figure 4B). Histidine and tryptophan induced GFP expression, methionine and glutamine reduced GFP expression, while the other amino acids had no effect.

Subsequently we determined the effects of supplemented TCA cycle intermediates and pyruvate on the *Phsp-4::GFP* ER stress response reporter strains of worms (Figure 5B). Citrate activated *Phsp-4::GFP* reporter gene expression, and there was also a strong trend (p = 0.05) for increased expression with alpha-ketoglutarate, while the other TCA cycle intermediates had no effect. Chelation of calcium or other metal ions is a possible mechanism as to how some of these compounds or their metabolites activate ER stress.

### Tryptophan induces the mitochondrial unfolded protein response

We further tested for activation of the mitochondrial unfolded protein response using *Phsp-6* and *Phsp-60* reporter strains and ethidium bromide treatment as the positive control. HSP-6 is the worm homolog of mammalian mitochondrial hsp-70, while HSP-60 is also localized to the mitochondrion. Both mitochondrial heat shock proteins play a role in the mitochondrial unfolded protein response, but this response is not always associated with longevity [58]. For the *Phsp-6::GFP* reporter strain we tested glutamine, histidine, serine, phenylalanine, and tryptophan (Figure 4C). We found phenylalanine and tryptophan to robustly increase expression, while the other amino acids did not increase expression. We further tested the effects of glutamine, histidine, phenylalanine, proline, serine, tryptophan, and tyrosine on GFP expression in the *Phsp-60::GFP* reporter strain (Figure 4D). We found only tryptophan to increase expression. There was also a strong trend for proline to increase expression (p = 0.06), while serine slightly decreased expression. Overall, most amino acids do not rely upon the mitochondrial unfolded protein response pathway for lifespan extension.

Next, we determined the effects of the TCA cycle intermediates and pyruvate on the mitochondrial unfolded protein response reporter strains. None of the metabolites affected expression of the *Phsp-6::GFP* reporter (Figure 5C), while pyruvate, succinate, and malate slightly decreased expression of the *Phsp-60::GFP* reporter strain (Figure 5D). Overall, the data suggest that TCA cycle intermediate supplementation does not require the mitochondrial unfolded protein response pathway for lifespan extension.

### Tryptophan metabolites nicotinic acid, nicotinamide, and picolinic acid induce both ER stress and mitochondrial unfolded protein responses

**Figure 6:**
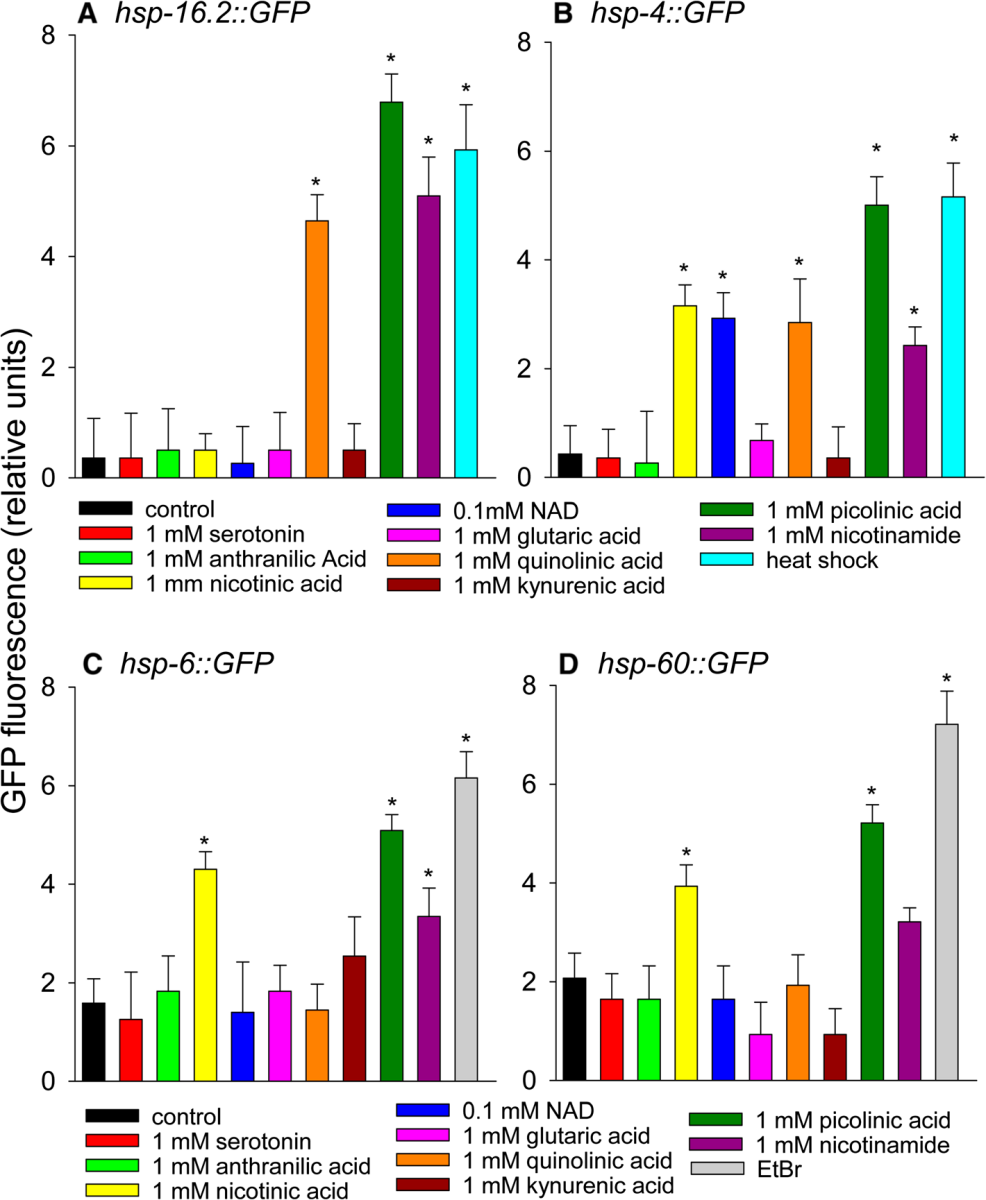
**The effects of tryptophan metabolites on heat shock, mitochondrial unfolded protein response, and ER stress response. (A)** *Phsp-16.2::GFP* **(B)** *Phsp-4::GFP* **(C)** *Phsp-6::GFP* **(D)** *Phsp-60::GFP* (*p < 0.05 vs. control). For panels A and B heat shock at 35°C for 2 hours was used as a positive control. For panels C and D 50 μg/ml ethidium bromide treatment for 2 days was used as a positive control (*p < 0.05 vs. control).

Since tryptophan activated expression of the ER stress response and 2 mitochondrial unfolded protein response reporter strains, we determined if one or more of its breakdown products or structurally related metabolites could also induce these responses. Therefore we added many of the tryptophan degradation products or tryptophan-related cellular metabolites including serotonin, anthranilic acid, nicotinic acid, nicotinamide, NAD, glutaric acid, kynurenic acid, quinolinic acid, and picolinic acid to the HSF-1 reporter strain, the ER stress reporter strain, and the 2 mitochondrial unfolded protein response reporter strains (Figure 6A-D). Strikingly, nicotinic acid activated expression of the same ER stress response and mitochondrial unfolded protein response reporters as tryptophan, while picolinic acid and nicotinamide activated expression of all 4 GFP reporter strains, although activation of *hsp-60::GFP* by nicotinamide was low (p = 0.07). Picolinic acid is an isomer of nicotinic acid and an important endogenous metal chelator [59]. Quinolinic acid induced expression of the 2 non-mitochondrial heat shock protein reporters. Therefore, we performed lifespan experiments adding 1 mM picolinic acid or 1 mM quinolinic acid to the culture medium (Additional file 6: Table S3). Picolinic acid addition showed a trend (p = 0.13) toward increased lifespan. In contrast, quinolinic acid addition decreased worm lifespan by 26%, as might be expected from its known neurotoxicity [60]. NAD precursors have previously been shown to induce the mitochondrial unfolded protein response [61]. Here, we added a 0.1 mM dose of NAD and found it to only activate expression of *hsp-4,* the marker of ER stress. Although tryptophan metabolic byproducts could contribute to the protective effects of supplemental tryptophan, others have shown data suggesting that tryptophan itself may be the protective metabolite in a *C. elegans* model of alpha-synuclein toxicity [27].

### Proline and tryptophan increase *C. elegans* thermotolerance

Amino acids could have induced expression of other heat shock or stress-inducible genes to extend lifespan not assayed in our reporter experiments described above. Therefore, we determined the effect of individual amino acid supplementation on the thermotolerance of *C. elegans*. We supplemented the growth medium with glutamine, histidine, proline, serine, or tryptophan and monitored the viability of the worms following transfer from 20°C to 35°C (Table 6 and Additional file 7: Figure S4A). Proline provided a 30% increase in thermotolerance and tryptophan provided a 10% increase in thermotolerance, while there was no significant effect of the other amino acids. The effect of proline is not too surprising given that proline stabilizes proteins and membranes and is overproduced to protect plants and some microorganisms from osmotic, salinity, and temperature stresses [62].

**Table 6:** **The effects of amino acids on** ***C. elegans*** **thermotolerance**

**Treatment**	**% of untreated mean survival**	**p-value**	**# of worms**	**Replicates**
5 mM histidine	102	0.494	229	2
1 mM tryptophan	110	<0.001	208	2
10 mM serine	102	0.794	196	2
5 mM proline	130	<0.001	200	2
5 mM glutamine	99	0.584	82	1

We next monitored the resistance to oxidative stress by monitoring the viability of worms following administration of paraquat (Additional file 7: Figure S4B). Paraquat is an inducer of superoxide production through redox cycling. None of the tested amino acids including proline, serine, histidine, tryptophan, or leucine significantly protected the worm viability against this stress, although there was a strong trend for protection with histidine (p = 0.08).

### Little effect of amino acid supplementation on Alzheimer’s amyloid-beta toxicity

**Figure 7:**
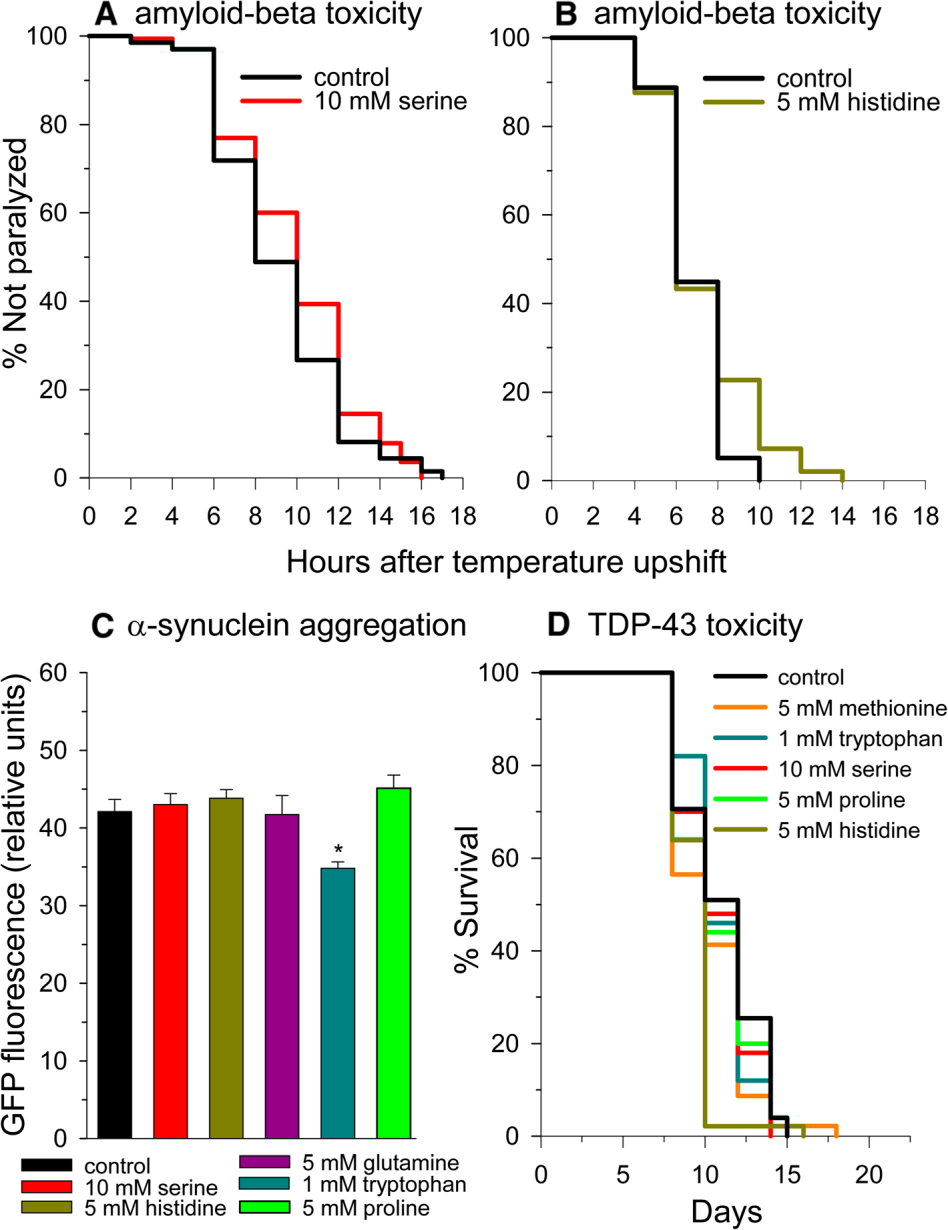
**Amino acids do not provide much protection in models of proteotoxicity. (A)** Serine and **(B)** histidine supplementation yield slight protection against muscle amyloid-beta toxicity. **(C)** Tryptophan supplementation decreases alpha-synuclein aggregate fluorescence. **(D)** Amino acid supplementation does not protect against TDP-43 toxicity.

Since many of the amino acids extended lifespan when they were supplemented to the culture medium, we also determined if individual amino acid supplementation could delay toxicity in *C. elegans* models of human neurodegenerative disorders. We first determined the effects of serine, histidine, proline, tryptophan, or methionine supplementation on the rate of paralysis development when the amyloid-beta peptide, which builds up in Alzheimer’s disease brain, is expressed in worm body wall muscle from a temperature-inducible promoter [63]. Overall, amino acid supplementation only had a minimal effect on the rate of paralysis. We found that serine (p = 0.07) (Figure 7A) or histidine (p = 0.05) (Figure 7B) supplementation gave strong trends to delay muscle paralysis, while no significant effects were found with proline, methionine, or tryptophan (Additional file 8: Table S4).

### Tryptophan protects from alpha-synuclein and polyglutamine toxicity

We next determined the effects of supplementation with histidine, proline, serine, or tryptophan on alpha-synuclein aggregation in worms expressing an alpha-synuclein-green fluorescent protein fusion (Figure 7C). Alpha-synuclein aggregates into Lewy bodies in the substantia nigra region of the brain in Parkinson’s disease. Tryptophan was highly protective giving a 17% reduction in aggregate fluorescence, while the other amino acids were without effect. We also tested the effects of tryptophan, serine, and proline on aggregates formed from the expression of a polyglutamine (Q35)–GFP fusion protein [64] (Additional file 9: Figure S5) as a model of the proteotoxicity that occurs in Huntington’s disease and other polyglutamine trinucleotide expansion disorders. We found a strong trend toward a decreased number of aggregates with the addition of tryptophan (p = 0.07), but no significant effect of the other two amino acids tested. It is not surprising that tryptophan supplementation was protective in these *C. elegans* proteotoxicity models as depletion of the TDO-2 enzyme that degrades tryptophan has been shown to increase worm tryptophan levels and be protective in several worm models of proteotoxicity [27]. It was also shown that tryptophan supplementation was able to increase the motility of Q40 polyglutamine expressing worms.

### Alpha-ketoglutarate, but not amino acids, protects *C. elegans* expressing TDP-43

Tar DNA-binding protein-43 (TDP-43) is mostly a nuclear protein under normal conditions, but cytoplasmic accumulation is associated with ALS and other neurodegenerative diseases. Overexpression of TDP-43 leads to toxicity in *C. elegans* and is used as an ALS model. We therefore tested if several amino acids including histidine, methionine, proline, serine, and tryptophan could alter the reduced lifespan of TDP-43 overexpressing worms (Figure 7D and Additional file 10: Table S5). However, none of these amino acids protected against the reduced lifespan, while histidine addition even further reduced the lifespan. But, we did find a protective effect of alpha-ketoglutarate supplementation in this model, supporting data from a study in which alpha-ketoglutarate in combination with other metabolites was protective in the SOD1-G93A mouse model of ALS [65].

### Serine and tryptophan partially block lifespan reduction in high glucose culture media

**Figure 8:**
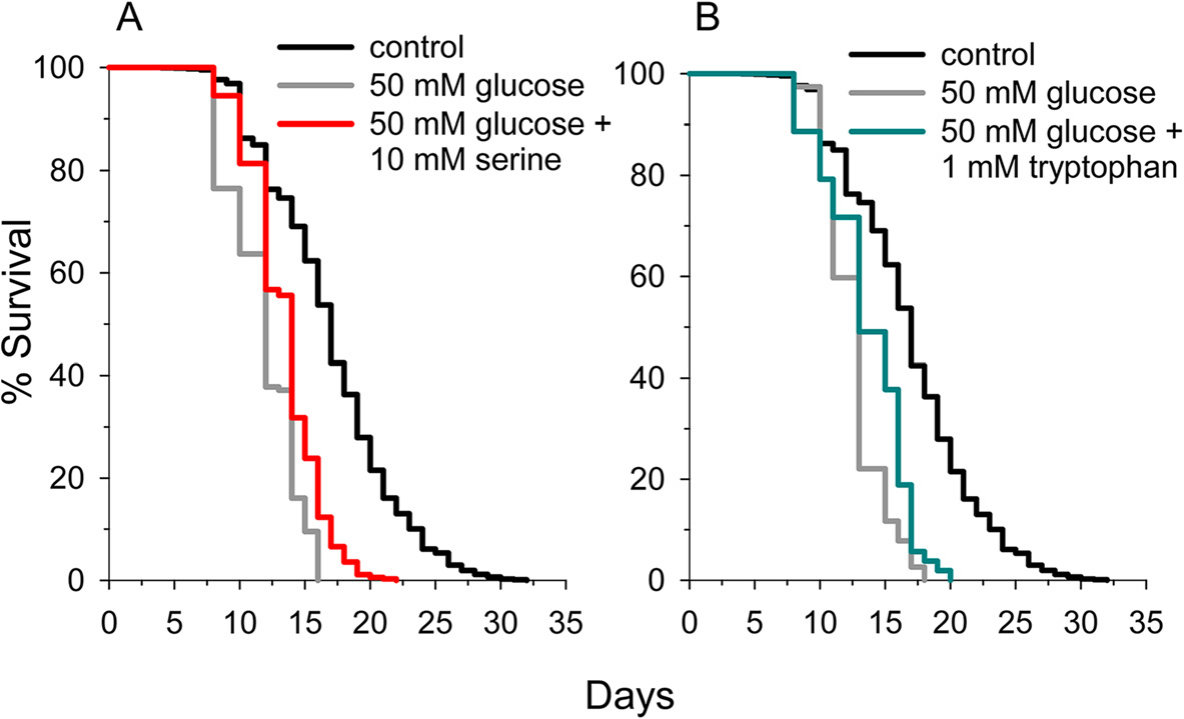
**Serine or tryptophan partially prevent high glucose from reducing** ***C. elegans*** **lifespan. (A)** Serine extends *C. elegans* lifespan in the presence of high glucose (p < 0.001). **(B)** Tryptophan extends *C. elegans* lifespan in the presence of high glucose (p < 0.001).

High glucose in the bloodstream is a marker of insulin resistance and diabetes and high levels of glucose in *C. elegans* culture medium is known to reduce lifespan [66,67]. Therefore high glucose supplementation has been used as a model of diabetes in *C. elegans*. Since alterations of amino acid levels also accompany diabetes, we determined if select amino acids could alter the decreased lifespan resulting from the addition of 50 mM glucose to the culture medium. We found glucose addition decreased lifespan by 30 percent and that serine (Figure 8A) or tryptophan (Figure 8B) supplementation partially blocked lifespan reduction. Histidine, proline, or tyrosine addition had no significant effect, while glutamine addition decreased lifespan to a greater extent than glucose by itself (Additional file 11: Table S6).

### Amino acid supplementation does not significantly alter *C. elegans* oxygen consumption and ATP levels

Since amino acid supplementation may transiently increase ROS production for the activation of SKN-1, we hypothesized that the added amino acids may be catabolized to increase mitochondrial ETC function. Therefore we measured worm oxygen consumption and ATP levels. However, we did not find significantly altered oxygen consumption or ATP levels following addition of the individual amino acids serine, proline, histidine, tryptophan, leucine, asparagine, or phenylalanine to the worms (Additional file 12: Figure S6). Therefore, the supplemented amino acids are either not being metabolized at a substantial rate or are being metabolized in replacement of respiratory substrates present in the *E. coli* food source preventing an overall increase in metabolic rate.

## Discussion

Individual supplementation with 18 of the 20 proteogenic amino acids at an optimal concentration increased the lifespan of *C. elegans*. This metabolite-induced lifespan extension was not specific for only amino acids as supplementation with TCA cycle intermediates and many different classes of cellular metabolites also led to significant lifespan extension, suggesting altered cellular metabolism or TCA cycle anaplerosis plays a prominent role. Of the 20 amino acids, serine and proline extended lifespan to the largest extent. Strikingly, these and other top amino acids did not significantly extend the lifespan of long-lived dietary restricted *eat-2* mutants. If similar effects occurred in humans, imbalanced amino acid diets could be developed as a dietary restriction mimetic to delay aging and aging-related disease. In this regard a methionine restricted diet has been used clinically to treat metabolic syndrome [68]. However, whether or not amino acid supplementation strategies will apply to higher eukaryotes remains to be determined as individual supplementation of methionine failed to extend the lifespan of fruit flies [24], while supplementation of methionine decreased the lifespan of mice [69].

### Individual amino acid supplementation may decrease the rate of translation to extend lifespan

Most of the well-studied longevity pathways in *C. elegans* such as DAF-16, SKN-1, AAK-2, SIR-2.1, and HIF-1 appear to be involved in the lifespan extension mediated by histidine, serine, and proline, with the exception of the HIF-1 pathway for proline. However, tryptophan extends lifespan in a SKN-1 and DAF-16 independent manner, although tryptophan did increase fluorescence of a DAF-16 reporter strain of worms. These different longevity pathways may converge to decrease the rate of translation by the ribosome for lifespan extension. A small to moderate amount of amino acid imbalance could hinder correct aminoacyl-tRNA synthetase charging of tRNAs slowing the rate of translation in a GCN-2 dependent manner to increase lifespan, while a higher amount of amino acid imbalance may lead to a toxic reduction in translation rates decreasing lifespan. Previously, a decreased rate of translation has been shown to be essential for lifespan extension in mitochondrial mutants [33], during TOR inhibition, or during dietary restriction [34]. Furthermore, long-lived *daf-2* mutants were also shown to have a reduced rate of translation [70]. Reduced mitochondrial translation causing mitonuclear protein imbalance has also been shown to lead to increased lifespan [71]. High rates of translation during aging may overwhelm protein chaperone systems resulting in proteotoxicity. Slowing translation or increasing heat shock protein activity may delay this proteotoxicity to increase longevity.

The branched chain amino acid leucine most strongly activates TOR kinase, which can increase the rate of translation and lead to decreased lifespan. Therefore, we were surprised to find that supplementation with 1 mM leucine greatly increased lifespan. In fact, leucine was the second most potent amino acid at this concentration for promoting longevity. Under our standard growth conditions TOR signaling may already be highly activated by amino acids present in the bacterial food source and so may not be able to be further activated. Instead, we hypothesize that leucine metabolism or the amino acid imbalance caused by excess leucine may have activated other signaling pathways leading to TOR inhibition. In this regard, the lack of lifespan extension by amino acids in *rsks-1* mutants suggests that individual amino acid supplementation inhibits TOR signaling to extend lifespan. Of the other two branched chain amino acids, valine supplementation showed similar lifespan trends as leucine, even though it is not a potent inducer of TOR activity, and isoleucine supplementation showed little effect on lifespan. At higher concentrations, branched chain amino acids did not affect lifespan as much as at the lower concentrations. Therefore, high levels of branched chain amino acids do not always directly correlate with lifespan extension. Consistent with this hypothesis, decreased levels of branched chain amino acids were found in long-lived Ames dwarf mice [72]. However, amino acid imbalance cannot explain the increased longevity conferred upon supplementation with many other diverse cellular metabolites. Therefore, TCA cycle anaplerosis leading to a reprogramming of mitochondrial metabolism may be the molecular mechanism responsible for lifespan extension.

### NAD precursors and tryptophan induce the ER stress response and the mitochondrial unfolded protein response

Another significant finding in this report is that supplementation with tryptophan, nicotinamide, nicotinic acid, or its isomer picolinic acid resulted in the activation of both the mitochondrial unfolded protein and the ER stress response pathways, while NAD addition induced only the ER stress response. In mouse muscle and heart, decreased nuclear NAD levels with aging cause a hypoxia inducible factor-1α (HIF-1α)-induced decrease in mitochondrial transcription leading to mitochondrial dysfunction that was reversed by supplementation with an NAD precursor [73]. In addition to reversing this aging-induced mitochondrial dysfunction, supplementing NAD precursors (or tryptophan) can further protect cells by inducing an ER stress response and a mitochondrial unfolded protein response, although it has already been shown that NAD precursors induce a mitochondrial unfolded protein response [61]. Picolinic acid is a strong metal chelator that is frequently taken with chromium as a possible treatment for metabolic syndrome, although the efficacy of this treatment has been questioned [74]. But picolinic acid itself has been shown to be neuroprotective [59]. It will be important to determine if the protective effects of picolinic acid are due to metal chelation and if picolinic acid, nicotinic acid, and nicotinamide also induce the ER stress response or mitochondrial unfolded protein response in mammalian cells.

### Is catabolism of amino acids important for their longevity effects?

The catabolism of supplemented amino acids likely mediates their effects on longevity, but there are likely exceptions to this rule. We were surprised to find no increase in oxygen consumption or ATP levels as markers of increased metabolism when amino acids were added to the culture medium. It was previously shown that proline catabolism and the resulting transient increase in ROS production by the electron transport chain were essential for its pro-longevity effects [26]. Most other amino acids may also be metabolized to emit a transient ROS signal leading to SKN-1 dependent lifespan extension. Possible evidence for this includes that enzymes for proline, tryptophan, phenylalanine, glutamine, and D-alanine degradation are upregulated in long-lived *daf-2* insulin receptor mutant worms [26], where SKN-1 is also activated [75].

However, alpha-ketoglutarate-mediated lifespan extension was shown to be independent of ROS production [48]. Therefore more downstream TCA cycle catabolites such as alpha-ketoglutarate, succinate, malate, and fumarate extend lifespan through SKN-1independent, but DAF-16 dependent mechanisms [53]. In addition, cysteine and tryptophan-induced lifespan extension were independent of SKN-1, so supplementation with these amino acids did not likely alter mitochondrial metabolism to increase ROS production. Consistent with this, knockdown of the tryptophan 2,3-dioxygenase (TDO-2) enzyme that degrades tryptophan led to increased lifespan and increased tryptophan levels, but no changes in the levels of many of the downstream metabolites of tryptophan degradation suggesting that increased tryptophan levels and not altered levels of the catabolic byproducts were responsible for the increased lifespan and protection from proteotoxicity [27].

Future studies will aim to determine the metabolic mechanisms through which amino acids activate SKN-1 activity and through which serine and histidine activate HIF-1. For example, enzyme knockdown studies using RNAi may be used to determine whether serine catabolism is required for serine-mediated lifespan extension, as serine can be directly deaminated to pyruvate, which extends lifespan [51]. However, serine can also be used as a one-carbon donor for methylation events such as histone methylation, which can alter gene expression patterns leading to increased longevity as well [76].

### TCA cycle metabolism and longevity

Different amino acids are catabolized and enter the TCA cycle through different intermediates of the cycle. We did not find that the amino acids yielding the greatest effects on lifespan were degraded through one common catabolic pathway. Additional file 13: Figure S7, Additional file 14 Figure S8, and Additional file 15: Figure S9 show the specific TCA cycle intermediate into which each of the amino acids is catabolized and how lifespan was affected by 1 mM, 5 mM, and 10 mM amino acid concentrations, respectively. As mentioned previously, the only trend in the data set is that amino acids broken down into alpha-ketoglutarate yielded larger than average lifespan extensions. However, this cannot explain the large lifespan extension induced by serine addition, which is catabolized to pyruvate. Surprisingly, we previously discovered that malate or fumarate supplementation increased total pyridine (NAD + NADH) nucleotide levels and also induced mild mitochondrial uncoupling increasing the NAD/NADH ratio, both of which may have been involved in the lifespan extension induced by these TCA cycle intermediates [53]. Catabolism of other metabolites may result in similar effects.

Knockdown of mitochondrial aconitase or a subunit of mitochondrial NAD-dependent isocitrate dehydrogenase increased lifespan in *C. elegans* [77]. The increased longevity of these mutants is likely due in part to decreased flux through this portion of the TCA cycle leading to increased NAD levels, which has been shown to extend lifespan [78]. The isocitrate dehydrogenase and alpha-ketoglutarate dehydrogenase enzymes reduce mitochondrial NAD to NADH. Citrate supplementation may not increase lifespan due to the increased flux through this portion of the TCA cycle leading to a decrease in NAD levels, although in the cytoplasm citrate is metabolized to acetyl-CoA that has been shown to inhibit autophagy, which can also prevent lifespan extension [79]. However, isocitrate supplementation would also not be expected to increase longevity as its normal metabolism is predicted to lower NAD levels. However, there is also an NADP-dependent isocitrate dehydrogenase isoform present that may help prevent declines in NAD levels and allow for isocitrate-mediated lifespan extension at least in a narrow range of concentrations.

It is possible that supplementation with amino acids broken down into alpha-ketoglutarate may extend lifespan by running isocitrate dehydrogenase in the opposite direction of its normal mode to oxidize NADH to NAD while alpha-ketoglutarate and carbon dioxide are metabolized into isocitrate. Some cancer cells have been shown to use this metabolism when oxidizing glutamine as a primary energy substrate [80]. The citrate produced from isocitrate is then exported to the cytoplasm where acetyl-CoA and oxaloacetate are formed by ATP citrate lyase. This metabolic flexibility in TCA cycle metabolism may be required for specific amino acid and TCA cycle metabolite-mediated longevity and is a hypothesis for future testing, as running the TCA cycle in the reverse of its normal direction was shown to be needed for malate and fumarate-mediated lifespan extension [53].

### Glycolysis as a lifespan shortening metabolic pathway in *C. elegans*

Since supplementation with glucose and other glycolytic precursors decrease lifespan in *C. elegans* as shown here and in [66,67], there are likely specific metabolic pathways such as glycolysis that decrease lifespan. We propose that many of the supplemented metabolites that increased lifespan are catabolized by mitochondria to increase TCA cycle metabolite levels. The DAF-16/FOXO longevity pathway has been shown to be activated by increased TCA cycle metabolite levels [53]. So the TCA cycle appears to be lifespan-extending metabolic pathway in *C. elegans*. In addition increased TCA cycle flux could also transiently increase mitochondrial ETC ROS production to activate the SKN-1 longevity pathway as previously suggested [26]. This metabolite oxidation for energy production would also decrease reliance on lifespan-shortening glycolysis as a source of mitochondrial respiratory substrates.

### Refinements in *C. elegans* culture media for lifespan experiments by limiting peptone levels and using heat-killed bacteria during adulthood

When performing lifespan experiments, sources of stress should be removed from the environment, so control animal lifespan is not limited. Unfortunately when working with *C. elegans*, this has proven difficult due to the slight toxicity of their live *E. coli* food source. We and others [55] have also found that the peptone present in nematode growth media (NGM) also induces a type of stress that decreases lifespan. It is unclear which component of peptone decreases lifespan as it contains not only proteolyzed proteins (amino acids and oligopeptides), but also fats, metals, salts, vitamins, and other compounds. Because of the slight toxicity associated with the use of peptone and live *E. coli*, we chose to perform *C. elegans* lifespan experiments using heat-killed *E. coli* in liquid S-medium, which lacks peptone. Due to partial degradation of one or more nutrients in the *E. coli* food source needed for larval development during the heat-killing treatment, it may be advantageous, especially in certain mutant backgrounds, to treat the worms with live *E. coli* during larval development to ensure adequate nutrition, and then switch them to heat-killed *E. coli* during adulthood to prevent the bacteria from metabolizing added nutrients. Further refinements in experimental methods will allow *C. elegans* to become an even more valuable model for investigating the effects of altered metabolism on lifespan.

## Conclusions

Individual amino acid supplementation increased the lifespan of *C. elegans* and increased stress resistance with serine, proline, and tryptophan showing the greatest effects. Many longevity pathways including DAF-16, SKN-1, AAK-2, SIR-2.1, GCN-2, heat shock, autophagy, DR, and inhibition of TOR signaling are involved in these protective effects. Anaplerosis and altered mitochondrial metabolism transiently increasing ROS production to activate SKN-1 appear to be involved in the longevity signaling. The exact pathways involved vary slightly from one amino acid to the next. For example, serine and histidine stimulated transcriptional activity of HIF-1, while 6 other amino acids did not. Likewise 8 lifespan-extending amino acids increased the transcriptional activity of SKN-1, but not tryptophan, cysteine, or the lifespan-decreasing amino acids phenylalanine and asparagine. Uniquely, tryptophan activated the ER stress response and mitochondrial unfolded protein response pathways and lifespan extension was independent of SKN-1 and DAF-16. Future experiments will aim to develop an improved axenic medium that can be used to determine the effects of amino acid restriction on the lifespan of *C. elegans*. Through the use of both amino acid supplementation and restriction, a diet may one day be developed that can substantially increase stress resistance and slow aging and the onset of aging-associated disorders.

## Methods

### *C. elegans* strains and maintenance

*C. elegans* strains were purchased from the *Caenorhabditis* Genetics Center (CGC, University of Minnesota) and were cultured in most experiments using standard *C. elegans* conditions at 20°C in liquid S medium, but in some experiments where indicated standard NGM agar media was used as in [81]. Lifespan assays were performed using the standard *C. elegans* N2 Bristol strain unless otherwise noted*.* Lifespan assays were also performed using the following strains: GR1307 [*daf-16(mgDf50*)], TG38 [*aak-2(gt33)*], DA1116 [*eat-2(ad1116)*], VC199 [*sir-2.1(ok434)*], RB1206 [*rsks-1(ok1255)*], RB967 [*gcn-2(ok871)*], RB579 [*Ife-2(ok306)*], TK22 [*mev-1(kn1)*], and CW152 [*gas-1(fc21)*]. Promoter::GFP gene expression reporter experiments were performed with the following strains: SJ4100 [*hsp-6::gfp*], SJ4058 [*hsp-60::gfp*], SJ4005 [*hsp-4::gfp],* CL2070 [*hsp-16-2::gfp + pRF4*], CL2166[*gst-4p::GFP::NLS*], ZG449[*egl-9(ia61) + nhr-57p::GFP + unc-119(+)*], CF1553[*sod-3p::GFP* + *rol-6*], and OP37[*unc-119(ed3)* + *pha-4::TY1::EGFP::3xFLAG* + *unc-119(+)*]. Disease and proteostasis protection assays were performed using the following strains: NL5901 [(*unc-54p::alpha-synuclein::YFP + unc-119(+))*], CL4176 [*smg*-1^ts^ [*myo*-3::Aβ_1–42_*long 3’-UTR*]], CL6049 [(*snb-1*::*hTDP-43* + *mtl-2*::*GFP*], and AM140 [*unc-54p::Q35::YFP*].

### Chemicals

L-Amino acids were purchased from Acros Organics and Research Products International Corp. Glycine was obtained from Fischer Chemical Company. D-amino acids were purchased from P212121, LLC. When no D or L isomer indication is present before the name of the amino acid (except for glycine), the L isomer was used. 5-fluoro-2’-deoxyuridine (FUdR) was purchased from Research Products International Corp. and Biotang, Inc. Sodium hydroxide (Fischer Scientific) was added to metabolite stock solutions to obtain a pH of 7.0.

### Lifespan analysis

Gravid *C. elegans* adults were bleach treated as previously described [53] to yield age-synchronized eggs. Almost all lifespan experiments were performed using liquid S-medium. Eggs suspended in S-media were placed in 3 μM transparent cell culture inserts (BD Falcon #353181) in 12-well microplates as first described in [82]. HT115 (DE3) *E. coli* were grown for 20 hours in 2 L flasks with LB media. The *E. coli* were then spun down, the supernatant poured off, and the pellet was frozen until use. The *E. coli* were de-thawed and heat-killed at 80°C for 1 hour with slight vibration using a Kendal model HB-S-23DHT ultrasonic cleaner. The *E. coli* pellets were then resuspended in an equal volume of S-medium. A 1.3 mL suspension of S-medium containing 9 × 10^9^ HT115 (DE3) *E. coli* per mL was placed in each well of a 12-well microplate. Bleach synchronized worm eggs were suspended at a concentration of 100–200 eggs/mL in the suspension of *E. coli* in S-medium. Lastly, a cell culture insert was placed in each well followed by 0.25 mL of the egg/bacterial suspension (25–50 eggs) into each insert (*n* = 3 wells per condition). Synchronized cultures of worms were placed on an orbital shaker at 135 rotations per minute at 20°C and monitored until they reached adulthood (~72 h), at which time FUdR was added to a final concentration of 400 uM. Worm viability was scored every two days. Worms that did not respond to repeated stimulus were scored as dead and those that contained internally hatched larvae were excluded. The culture media containing *E. coli* in the 12-well plates below each culture insert (>80% of total culture media volume) was changed every 3 days. Lifespan analysis using the CL6049 [*snb-1*::*hTDP-43* + *mtl-2::gfp*] strain was performed in the same manner except worm viability was counted every day.

### High glucose lifespan assays

We performed *C. elegans* lifespan assays as described above with the addition of 50 mM glucose to the culture medium. Worms were scored for viability every day. The culture media in the 12-well plates was changed every two days.

### RNAi feeding experiments

The *E. coli skn-1* and *bec-1* RNAi clones from the Ahringer *C. elegans* RNAi library (Source BioScience LifeSciences) were grown 16 hours and then 1 mM IPTG was administered to the *E. coli* for the last 4 hours of growth to induce expression of the double strand RNA. Lifespan experiments were performed using live instead of heat-killed bacteria. The culture media in the 12-well plates was changed daily instead of every 3 days to decrease the chance of *E. coli* metabolism depleting the culture level of the supplemented amino acid.

### GFP reporter strains

GFP fluorescence of *C. elegans* populations was assayed using a Biotek Synergy 2 multi-mode microplate reader. Strains were age synchronized and cultured in 12-well microplates as described above. At the L3 stage of larval development, worms were treated with amino acids or other metabolites. Following 24 hours of treatment, worms were washed 3 times in S-medium and approximately 400 worms in 200 μL were added to each well of a clear, flat-bottom 96-well plate, and GFP fluorescence was measured using 485/20 nm excitation and 528/20 nm emission filters (a minimum of n = 8 per treatment group).

### Microscopy and quantification

Worms used for microscopy were anesthetized using 1 mM levamisole and transferred to agar pads with glass coverslips and analyzed using an EVOS fluorescence microscope. Comparable results were established in the absence of levamisole. Approximately 20 worms per condition were used and all experiments were repeated at least three times. ImageJ™ software was used to quantify pixel intensities.

### Thermotolerance assays

A synchronized population of N2 *C. elegans* eggs were placed on treated or non-treated NGM agar plates and allowed to hatch at 20°C. At the L4 stage of development animals were transferred to a 35°C incubator. Survival was scored as the number of worms responsive to gentle prodding with a worm pick.

### Aß-mediated paralysis assays

Paralysis assays were carried out as outlined in [63]. Briefly, second generation synchronized gravid *C. elegans* strain CL4176 grown at 16°C were placed on treated or untreated 6 cm NGM agar plates and allowed to lay eggs for 2 hours. After that, adults were removed and plates were placed in a 16°C incubator for 48 hours. Then plates were transferred to a 25°C incubator. Scoring for paralyzed worms began 20 hours after temperature upshift. Animals were scored for movement every two hours. Worms were considered paralyzed if they could not complete a full body movement after stimulation with a worm pick.

### Alpha-synuclein aggregation assays

Eggs were collected from the NL5901 strain of *C. elegans* following treatment with alkaline bleach and placed in 12 well cell culture inserts as described above, with or without amino acid treatment. Following 2 days of treatment, 400 μM FUdR was added to the inserts to prevent progeny. On day 8 worms were washed 3 times with M9 media and either placed on 1% agarose pads to slow movement or immobilized with 1 mM levamisole. Quantification of the number of inclusions expressing alpha-synuclein-YFP was measured using an EVOS fluorescence microscope. Foci larger than 2 μm^2^ were counted for each group (n = 30). Image analysis was performed using ImageJ™ software and the assay was completed at least 3 times [83]. Statistical analysis was completed using GraphPad Prism software and calculation of statistical significance between groups was carried out using Student’s *t*-test.

### Polyglutamine aggregation assays

AM140 [*unc-54p::Q35::YFP*] worms were synchronized and placed onto NGM agar plates supplemented with either 1 mM tryptophan, 10 mM serine, or 5 mM proline. Plates were seeded with 90% heat-killed OP50 *E. coli* and 10% live OP50 *E. coli*. Images were taken at day 3 of adulthood. Progeny were avoided by picking daily after day 1. Aggregates were scored for 50 worms per condition in independent biological duplicates.

### Medium oxygen measurements

Medium oxygen measurements were acquired using flat bottom 96-well PreSens OxoPlates according to the manufacturer’s guidelines. Worm eggs from bleach-killed populations were placed in 12-well cell culture plates in 1 mL of S-medium with live HT115 (DE3) bacteria and a supplemented amino acid. At the L4 developmental stage, worms were washed 3 times with S-media and concentrated to approximately 10 worms/μL. 200 μL of each treatment group was placed into each well of an OxoPlate in replicates of 3–4. Oxygen concentration was measured using an excitation filter of 540/25 nm and emission filters of 590/20 nm (indicator) and 620/40 nm (reference) using a Biotek Synergy 2 microplate reader. Oxygen measurements were normalized to worm protein as in [53].

### ATP measurements

Bleach synchronized eggs were grown in liquid S medium in the absence or presence of an amino acid in the presence of live HT115(DE3) *E. coli*. At the L4 stage of larval development the worms were washed 3 times with S-medium to free them of bacteria and then lysed by repeated freeze-thaw as in [53]. ATP levels were measured using CellTiter-Glo (Promega) according to the manufsacturer’s directions and normalized to worm protein.

### Statistical analysis

Kaplan-Meier survival analysis and log-rank tests were performed using Sigmaplot version 11.0. Student’s t-tests were used in other analyses.

## Additional files

Additional file 1: Table S1. The effects of amino acid supplementation on *C. elegans* lifespan. Additional file 2: Figure S1. Example lifespan curves for several amino acids that strongly extended lifespan in *C. elegans*. (A) serine, (B) proline, (C) tryptophan, and (D) histidine. Concentrations chosen for display were those that stimulated lifespan extension to the greatest extent (log rank p < 0.001). Additional file 3: Figure S2. Supplementation of several D-amino acids found endogenously in *C. elegans* extends lifespan. (A) D-alanine, B) D-aspartate, or C) D-glutamate extended lifespan at one or more of the concentrations tested (log rank p < 0.05), while (D) D-serine supplementation did not extend lifespan at any of the concentrations tested. Additional file 4: Figure S3. The effect of amino acids on the fluorescence of a *pha-4p::gfp:pha-4* reporter strain of *C. elegans* (* p < 0.05)*.*
Additional file 5: Table S2. The effects of sugars and other metabolites lacking nitrogen on *C. elegans* lifespan. Additional file 6: Table S3. The effects of nitrogen containing metabolites on *C. elegans* lifespan. Additional file 7: Figure S4. (A) Proline or tryptophan supplementation increases thermotolerance in *C. elegans*. (log rank p < 0.001) Serine, histidine, or glutamine supplementation did not significantly affect thermotolerance. (B) Amino acid supplementation did not significantly delay paraquat-induced toxicity. However, there was a strong trend toward protection with histidine (p = 0.08), but no effect with serine, proline, tryptophan, or leucine. Additional file 8: Table S4. The effects of amino acids on amyloid-beta-induced muscle paralysis. Additional file 9: Figure S5. Tryptophan slightly decreased polyglutamine aggregates in *C. elegans*. The GFP fluorescence of five worms placed side by side are shown in each photo. There were on average 7 less aggregates in tryptophan treated worms than in controls (p = 0.07). Additional file 10: Table S5. The effects of amino acids or alpha-ketoglutarate on lifespan in human TDP-43 transgenic *C. elegans*. Additional file 11: Table S6. The effects of amino acids on *C. elegans* lifespan in the presence of 50 mM glucose. Additional file 12: Figure S6. Amino acid supplementation did not significantly alter *C. elegans* oxygen consumption or ATP levels. A) The amount of oxygen in the medium following a 30 minute incubation in the well of an Oxoplate. B) ATP levels. Additional file 13: Figure S7. Metabolism and effects on lifespan of a 1 mM dose of amino acids. A diagram of the TCA cycle metabolites to which the 20 amino acids are catabolized is shown. It is also shown how supplementation of a 1 mM concentration of the amino acids or some of the TCA cycle metabolites affected *C. elegans* lifespan. Additional File 14: Figure S8. Metabolism and effects on lifespan of a 5 mM dose of amino acids. A diagram of the TCA cycle metabolites to which the 20 amino acids are catabolized is shown. It is also shown how supplementation of a 5 mM concentration of the amino acids or some of the TCA cycle metabolites affected *C. elegans* lifespan. Additional file 15: Figure S9. Metabolism and effects on lifespan of a 10 mM dose of amino acids. A diagram of the TCA cycle metabolites to which the 20 amino acids are catabolized is shown. It is also shown how supplementation of a 10 mM concentration of the amino acids or some of the TCA cycle metabolites affected *C. elegans* lifespan.
