# Supplementary material for: Mechanisms of amino acid-mediated lifespan extension in Caenorhabditis elegans
Source: BMC Genet. 2015 Feb 3;16(1):8. doi: 10.1186/s12863-015-0167-2 (PMC4328591; doi:10.1186/s12863-015-0167-2)
Supplement: Additional file 1: Table S1. — The effects of amino acid supplementation on C. elegans lifespan. [file 12863_2015_167_MOESM1_ESM.pdf]

Table S1. The effect of amino acid supplementation on *C. elegans* lifespan

Strain	Concentration	Amino acid	% of control mean lifespan	*n*	# of worms	*p*-value
N2	1 mM	alanine	108	3	264	0.015
N2	5 mM		104	3	277	0.305
N2	10 mM		111	3	274	0.003
N2	1 mM	arginine	108	4	428	0.007
N2	5 mM		111	4	412	<0.001
N2	10 mM		115	4	438	<0.001
N2	1 mM	asparagine	101	3	350	0.331
N2	5 mM		105	3	319	0.032
N2	10 mM		75	3	297	<0.001
N2	1 mM	aspartate	103	3	477	0.150
N2	5 mM		100	3	495	0.052
N2	10 mM		94	3	544	0.001
N2	1 mM	cysteine	109	2	200	0.029
N2	5 mM		116	2	190	<0.001
N2	10 mM		102	2	195	0.513
N2	1 mM	glutamate	114	2	156	0.005
N2	5 mM		111	2	191	0.034
N2	10 mM		92	2	172	0.005
N2	1 mM	glutamine	115	2	184	0.002
N2	5 mM		116	2	196	0.002
N2	10 mM		94	2	214	0.001
N2	1 mM	glycine	110	3	321	0.008
N2	5 mM		103	3	298	0.538
N2	10 mM		96	3	327	0.090
N2	1 mM	histidine	104	3	338	0.155
N2	5 mM		109	3	269	<0.001
N2	10 mM		112	3	282	<0.001
N2	1 mM	isoleucine	103	3	344	0.079
N2	5 mM		103	3	290	0.066
N2	10 mM		103	3	360	0.033
N2	1 mM	leucine	116	2	265	<0.001
N2	5 mM		106	2	238	0.042
N2	10 mM		107	2	240	0.032
N2	1 mM	lysine	107	4	562	<0.001
N2	5 mM		108	4	583	<0.001
N2	10 mM		106	4	547	<0.001
N2	1 mM	methionine	104	2	160	0.094
N2	5 mM		108	2	162	0.002
N2	10 mM		114	2	148	<0.001
N2	1 mM	phenylalanine	97	2	167	0.491
N2	5 mM		92	2	165	<0.001
N2	10 mM		88	2	164	<0.001

N2	1 mM	proline	117	3	369	<0.001
N2	5 mM		119	3	322	<0.001
N2	10 mM		118	3	329	<0.001
N2	1 mM	serine	108	5	495	<0.001
N2	5 mM		118	5	501	<0.001
N2	10 mM		122	5	607	<0.001
N2	1 mM	threonine	100	3	317	0.901
N2	5 mM		103	3	326	0.179
N2	10 mM		108	3	279	<0.001
N2	1 mM	tryptophan	114	3	289	<0.001
N2	5 mM		106	3	234	0.003
N2	10 mM		101	3	203	0.643
N2	1 mM	tyrosine	110	2	198	<0.001
N2	5 mM		105	2	186	0.001
N2	10 mM		102	2	168	0.014
N2	1 mM	valine	113	3	338	0.155
N2	5 mM		108	3	269	<0.001
N2	10 mM		99	3	282	<0.001
