# Supplementary figures and images for: Mechanisms of amino acid-mediated lifespan extension in Caenorhabditis elegans
Source: BMC Genet. 2015 Feb 3;16(1):8. doi: 10.1186/s12863-015-0167-2 (PMC4328591; doi:10.1186/s12863-015-0167-2)

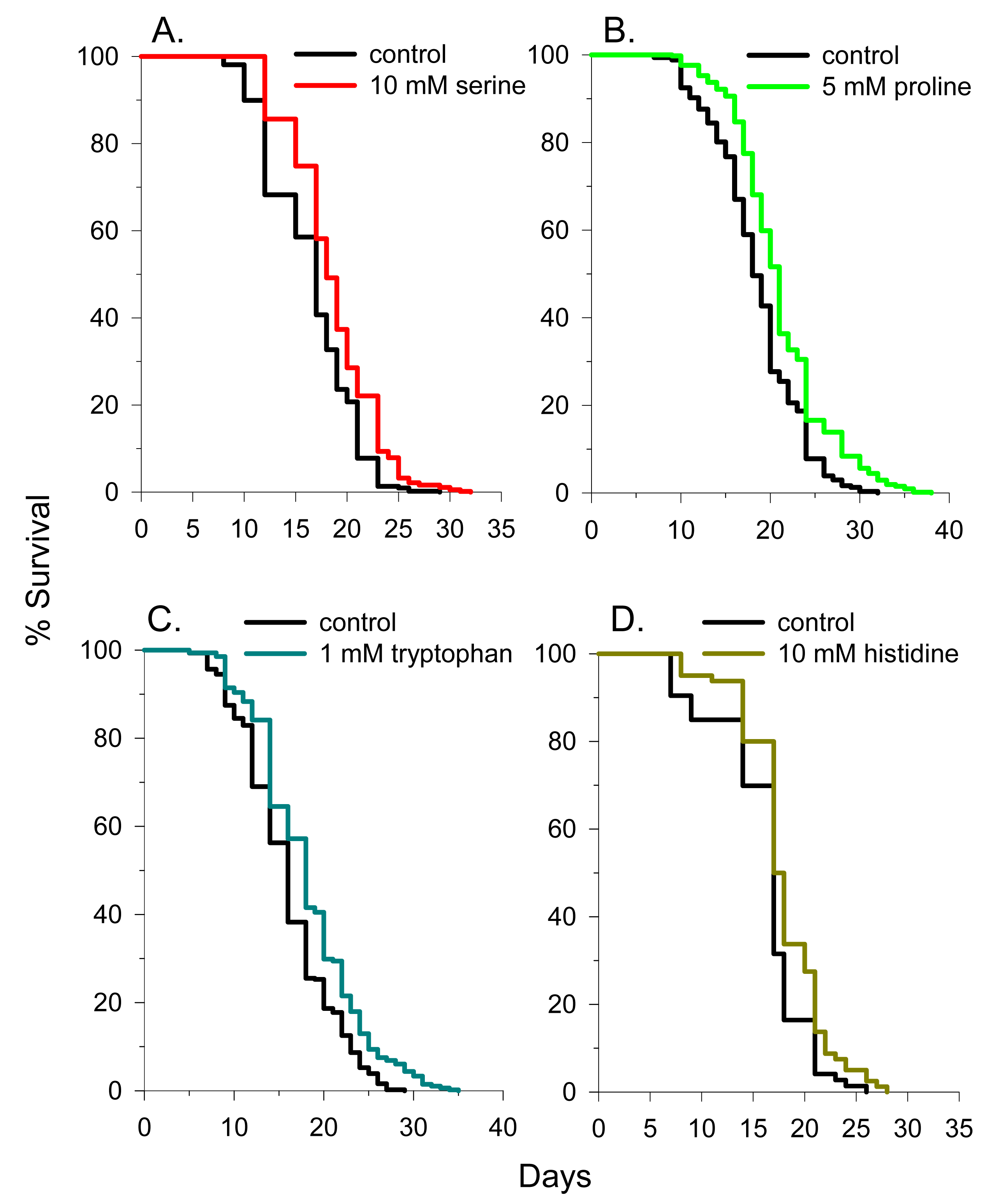

Supplement: Additional file 2: Figure S1. — Example lifespan curves for several amino acids that strongly extended lifespan in C. elegans. (A) serine, (B) proline, (C) tryptophan, and (D) histidine. Concentrations chosen for display were those that stimulated lifespan extension to the greatest extent (log rank p < 0.001). [file 12863_2015_167_MOESM2_ESM.tiff]

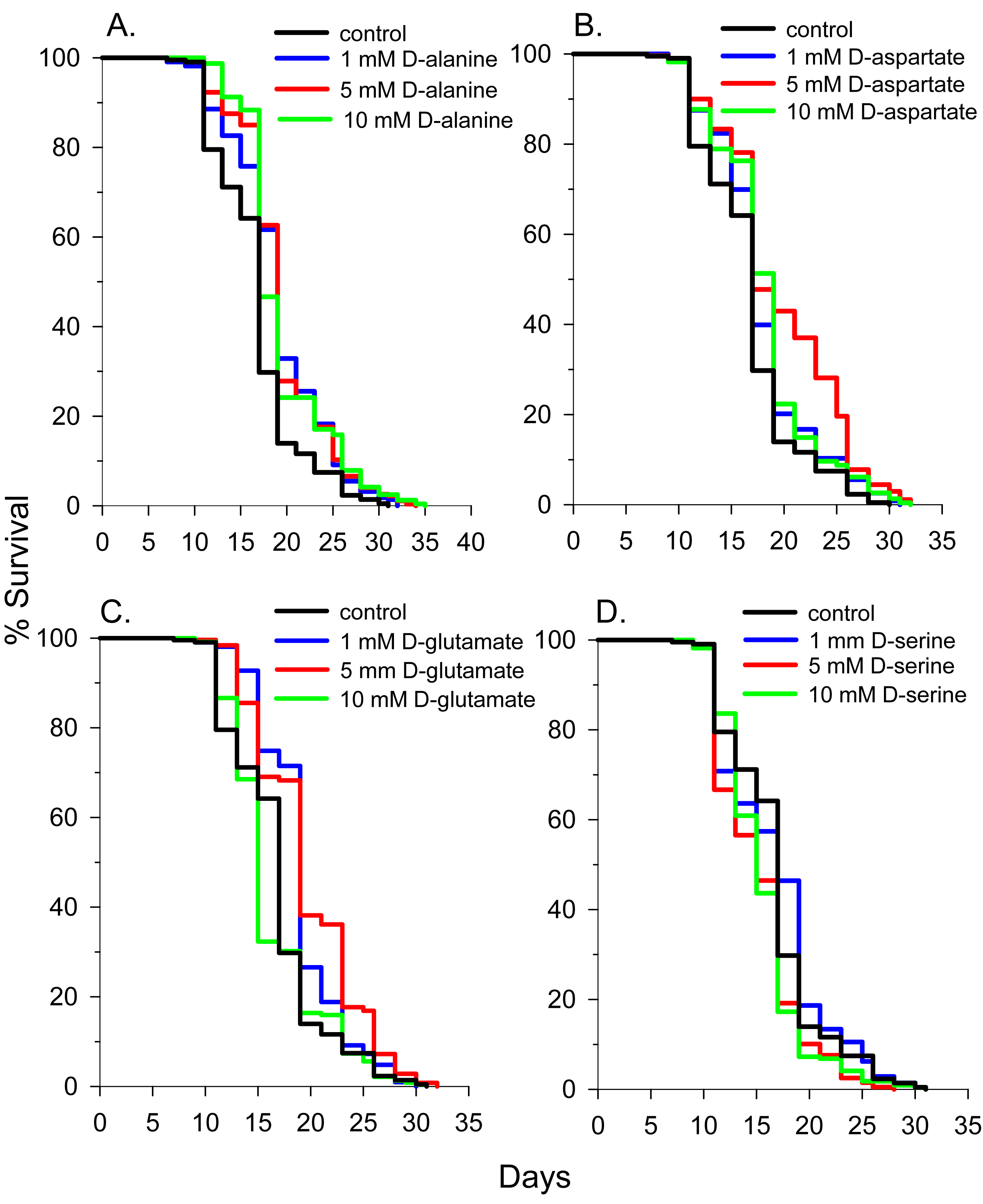

Supplement: Additional file 3: Figure S2. — Supplementation of several D-amino acids found endogenously in C. elegans extends lifespan. (A) D-alanine, B) D-aspartate, or C) D-glutamate extended lifespan at one or more of the concentrations tested (log rank p < 0.05), while (D) D-serine supplementation did not extend lifespan at any of the concentrations tested. [file 12863_2015_167_MOESM3_ESM.tiff]

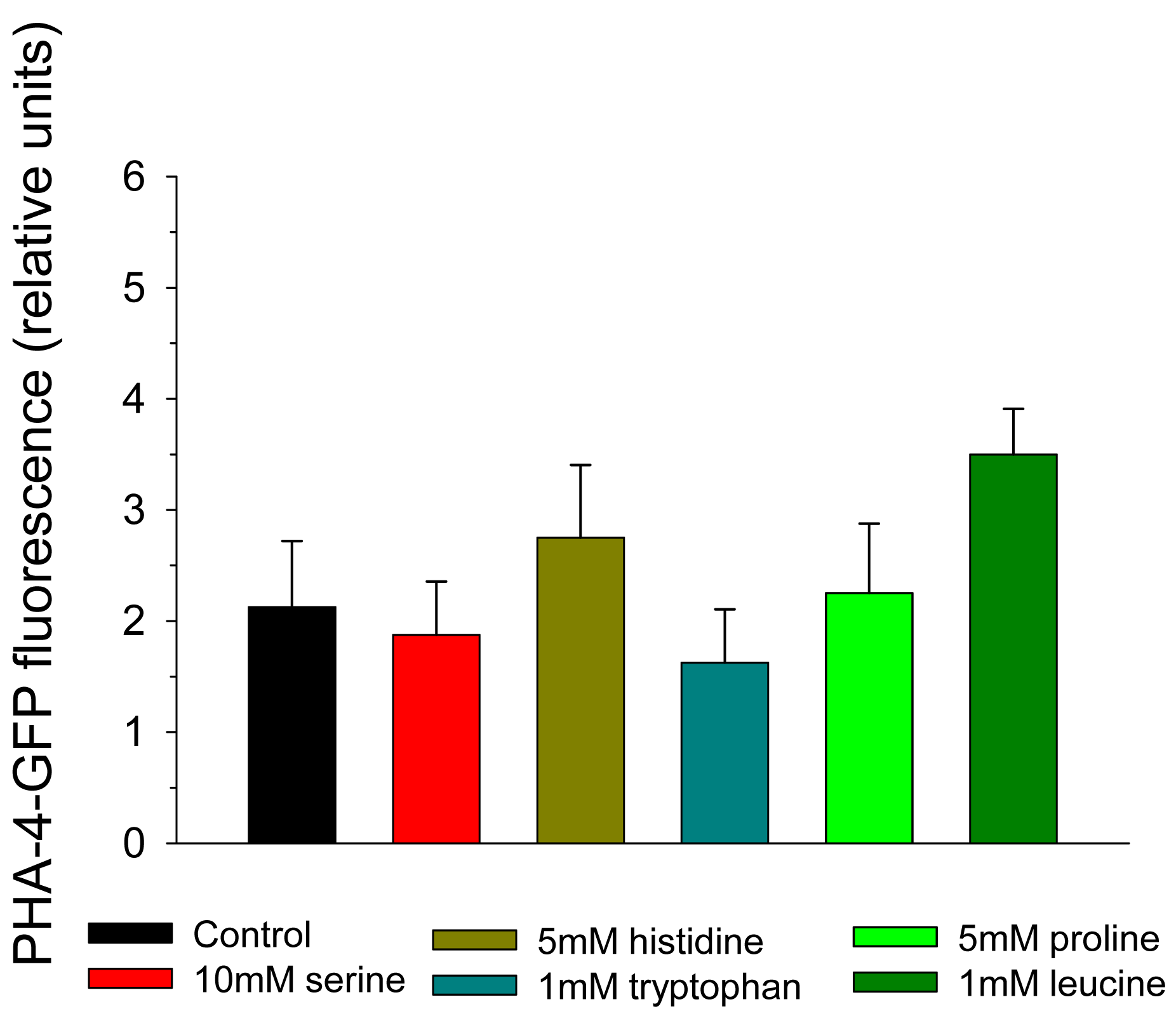

Supplement: Additional file 4: Figure S3. — The effect of amino acids on the fluorescence of a pha-4p::gfp:pha-4 reporter strain of C. elegans (* p < 0.05). [file 12863_2015_167_MOESM4_ESM.tiff]

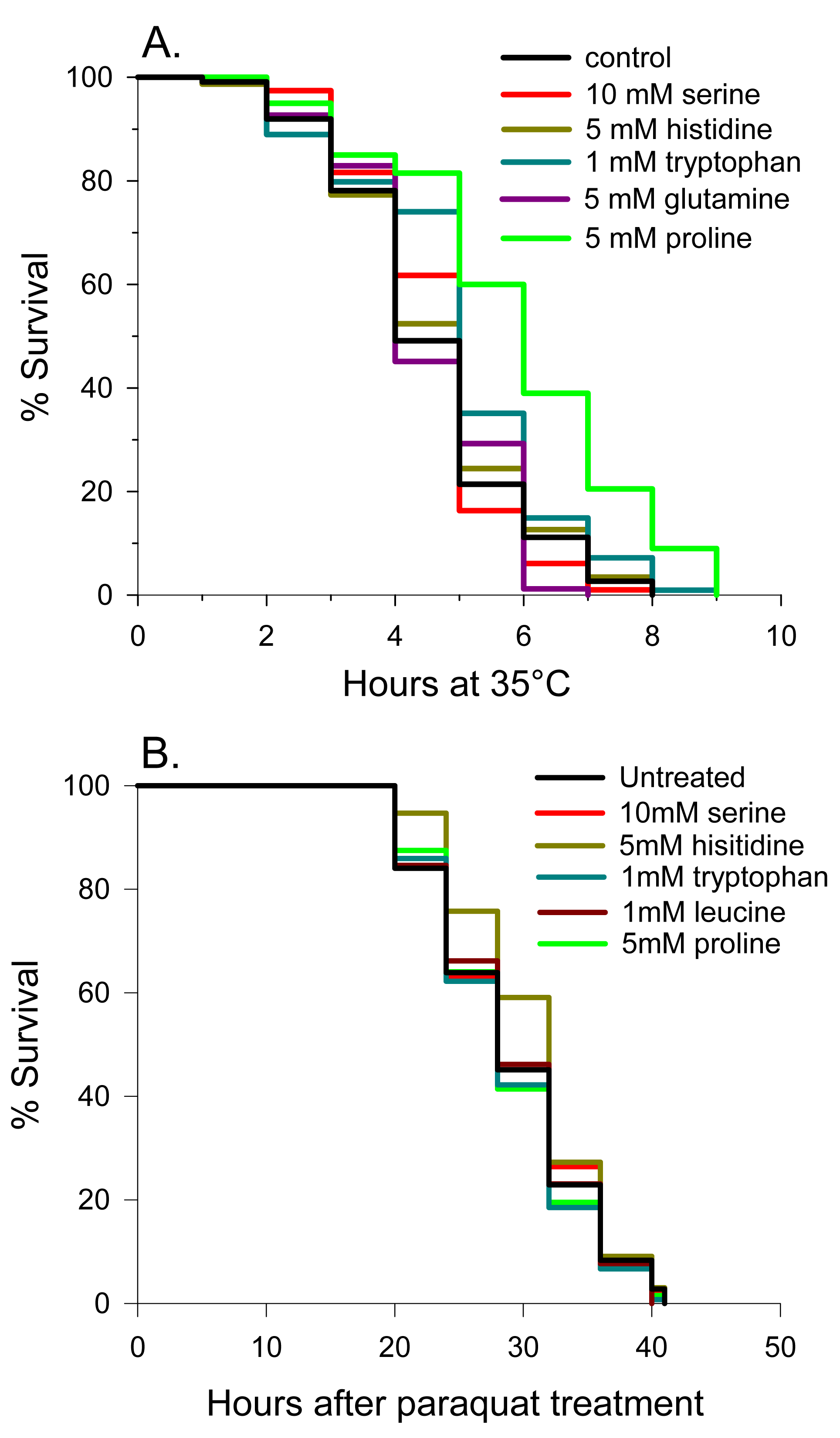

Supplement: Additional file 7: Figure S4. — (A) Proline or tryptophan supplementation increases thermotolerance in C. elegans. (log rank p < 0.001) Serine, histidine, or glutamine supplementation did not significantly affect thermotolerance. (B) Amino acid supplementation did not significantly delay paraquat-induced toxicity. However, there was a strong trend toward protection with histidine (p = 0.08), but no effect with serine, proline, tryptophan, or leucine. [file 12863_2015_167_MOESM7_ESM.tiff]

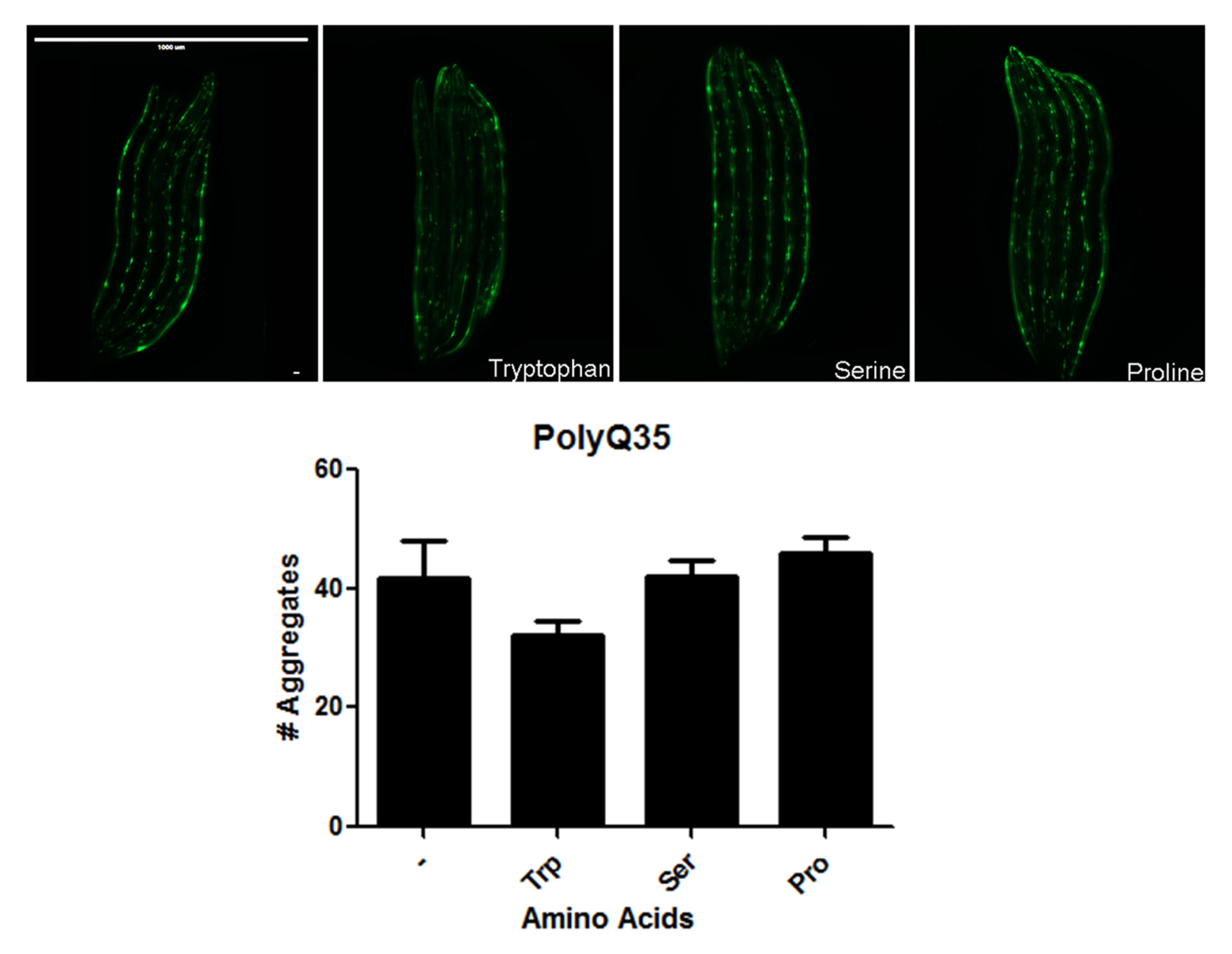

Supplement: Additional file 9: Figure S5. — Tryptophan slightly decreased polyglutamine aggregates in C. elegans. The GFP fluorescence of five worms placed side by side are shown in each photo. There were on average 7 less aggregates in tryptophan treated worms than in controls (p = 0.07). [file 12863_2015_167_MOESM9_ESM.tiff]

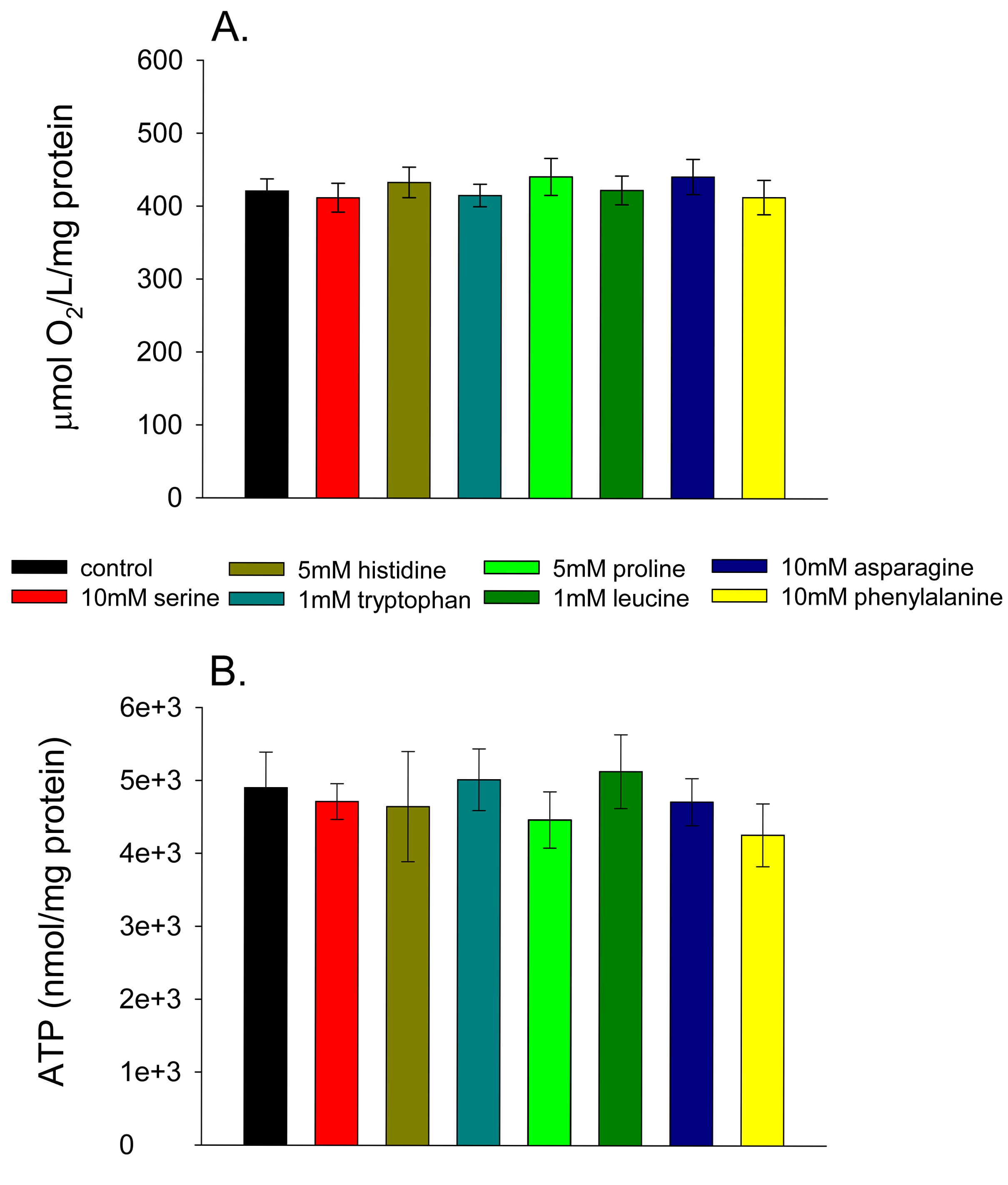

Supplement: Additional file 12: Figure S6. — Amino acid supplementation did not significantly alter C. elegans oxygen consumption or ATP levels. A) The amount of oxygen in the medium following a 30 minute incubation in the well of an Oxoplate. B) ATP levels. [file 12863_2015_167_MOESM12_ESM.tiff]

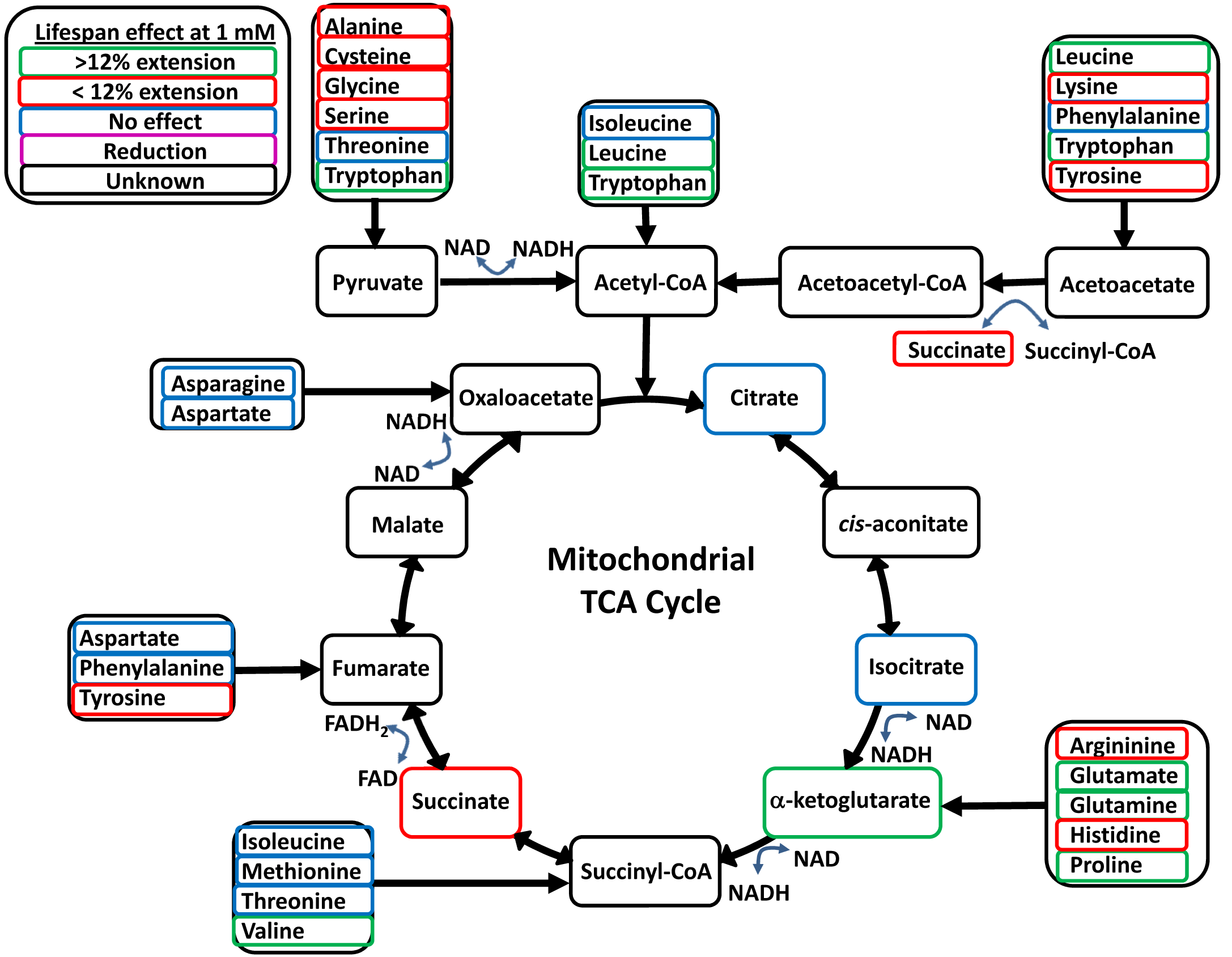

Supplement: Additional file 13: Figure S7. — Metabolism and effects on lifespan of a 1 mM dose of amino acids. A diagram of the TCA cycle metabolites to which the 20 amino acids are catabolized is shown. It is also shown how supplementation of a 1 mM concentration of the amino acids or some of the TCA cycle metabolites affected C. elegans lifespan. [file 12863_2015_167_MOESM13_ESM.tiff]

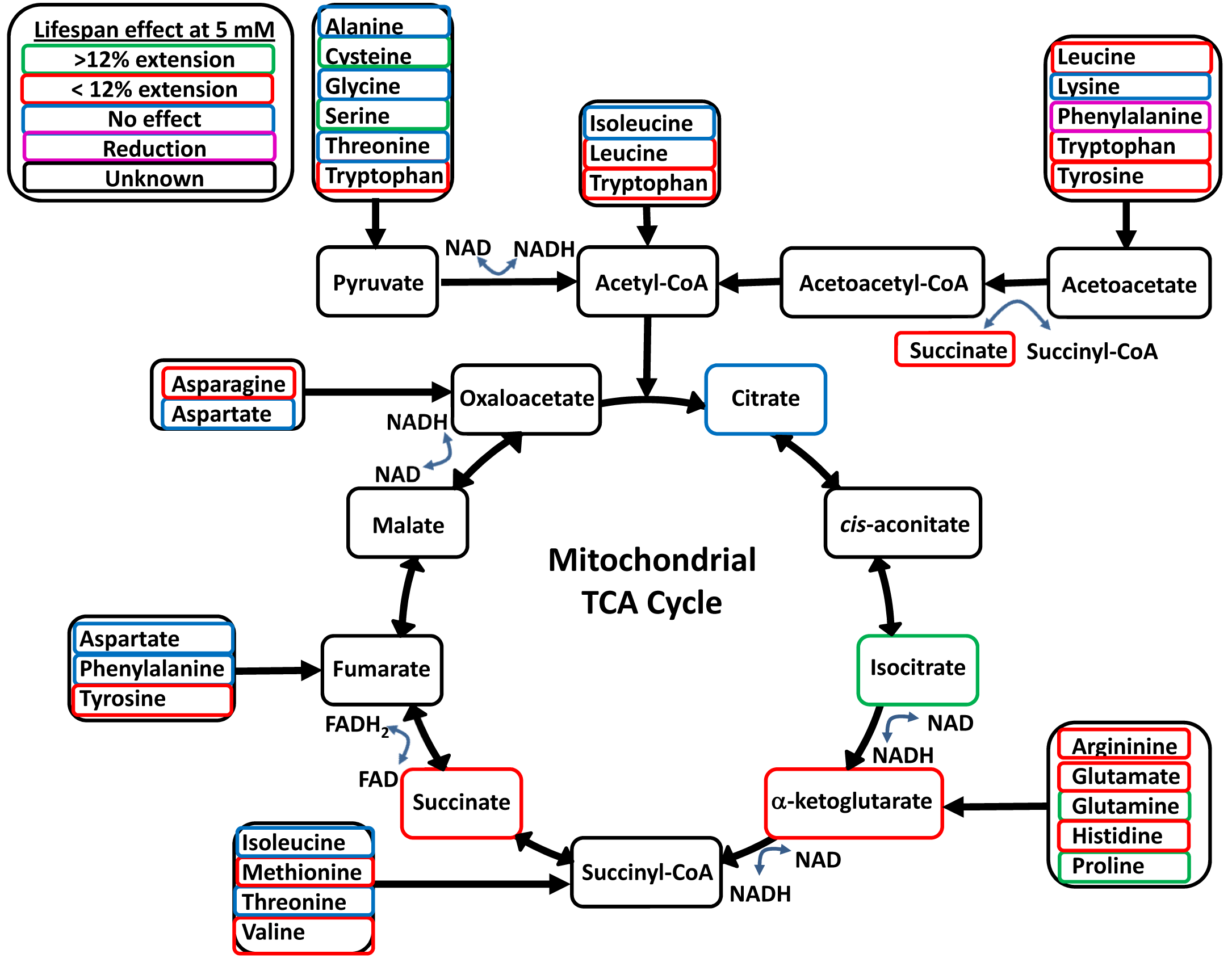

Supplement: Additional File 14: Figure S8. — Metabolism and effects on lifespan of a 5 mM dose of amino acids. A diagram of the TCA cycle metabolites to which the 20 amino acids are catabolized is shown. It is also shown how supplementation of a 5 mM concentration of the amino acids or some of the TCA cycle metabolites affected C. elegans lifespan. [file 12863_2015_167_MOESM14_ESM.tiff]

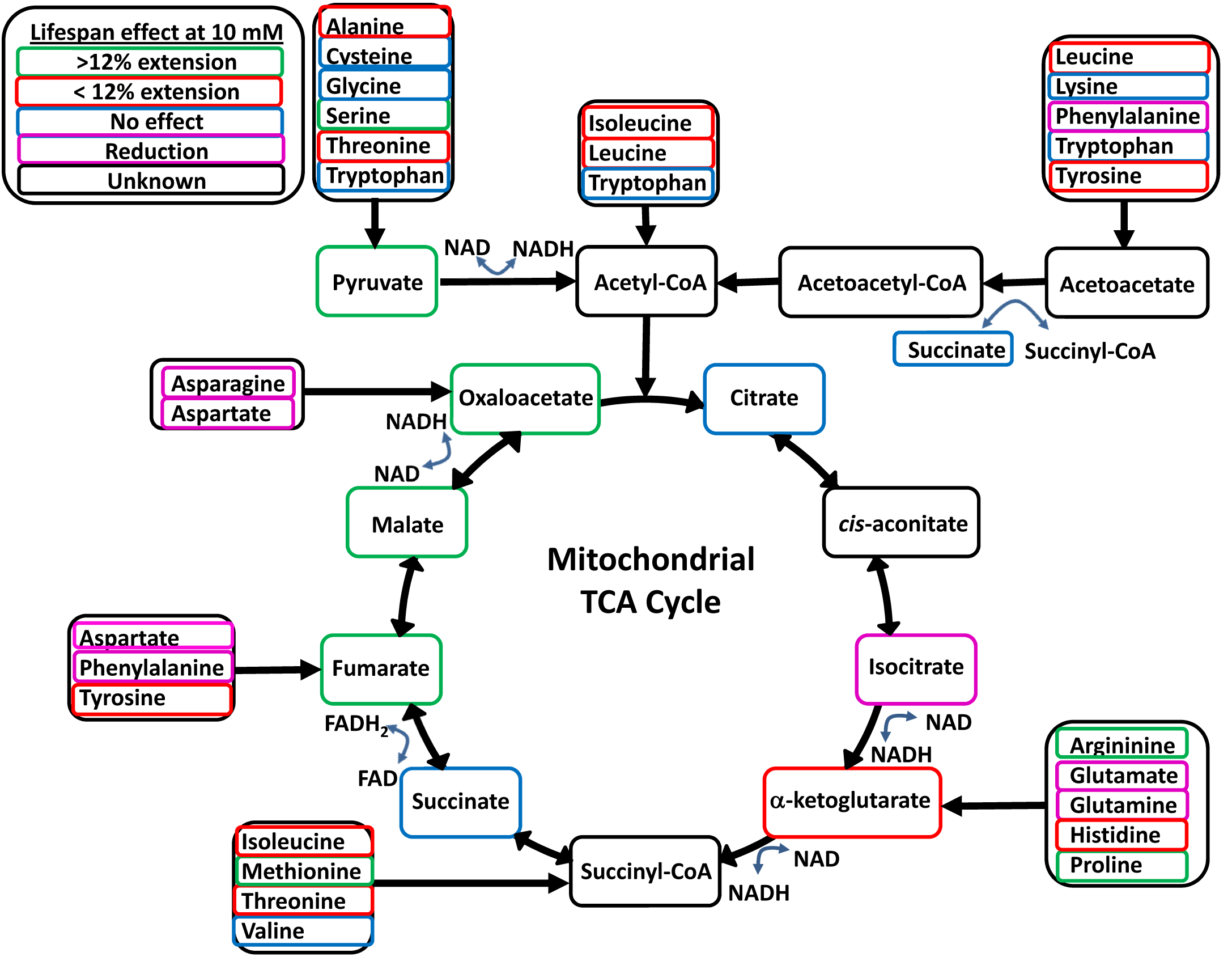

Supplement: Additional file 15: Figure S9. — Metabolism and effects on lifespan of a 10 mM dose of amino acids. A diagram of the TCA cycle metabolites to which the 20 amino acids are catabolized is shown. It is also shown how supplementation of a 10 mM concentration of the amino acids or some of the TCA cycle metabolites affected C. elegans lifespan. [file 12863_2015_167_MOESM15_ESM.tiff]
