# Supplementary material for: Mechanisms of amino acid-mediated lifespan extension in Caenorhabditis elegans
Source: BMC Genet. 2015 Feb 3;16(1):8. doi: 10.1186/s12863-015-0167-2 (PMC4328591; doi:10.1186/s12863-015-0167-2)
Supplement: Additional file 5: Table S2. — The effects of sugars and other metabolites lacking nitrogen on C. elegans lifespan. [file 12863_2015_167_MOESM5_ESM.pdf]

Table S2. The effect of sugars and other metabolites lacking nitrogen on *C. elegans* lifespan

**treatment**	**% of N2 control mean lifespan**	**p-value**	**# of worms**	**replicates**
1 mM ribose	109	<0.001	333	3
5 mM ribose	109	<0.001	345	3
10 mM ribose	101	0.726	377	3
10 mM glycerol	121	<0.001	134	1
1 mM dihydroxyacetone	77	<0.001	275	2
10 mM dihydroxyacetone	75	<0.001	269	2
1 mM glyceraldehyde	74	<0.001	113	1
10 mM glyceraldehyde	87	<0.001	186	1
1 mM inositol	103	0.0397	220	1
10 mM inositol	117	<0.001	235	1
10 mM xylose	106	0.038	133	1
1 mM galactose	101	0.887	296	2
10 mM galactose	106	0.0349	250	2
1 mM gluconate	108	0.0263	127	1
5 mM gluconate	117	<0.001	124	1
10 mM gluconate	116	<0.001	120	1
1 mM glucuronolactone	102	0.717	290	1
5 mM glucuronolactone	103	0.829	300	1
10 mM glucuronolactone	103	0.831	310	1
1 mM propionate	85	<0.001	104	1
5 mM propionate	87	<0.001	106	1
10 mM propionate	82	<0.001	110	1
1 mM fructose	96	0.010	251	2
10 mM fructose	86	<0.001	233	2
1 mM phosphoenolpyruvate	112	<0.001	180	1
5 mM phosphoenolpyruvate	112	<0.001	170	1
10 mM phosphoenolpyruvate	108	0.002	149	1
0.01 mM caprylate	101	0.356	192	2
0.1 mM caprylate	107	0.019	165	2
1 mM caprylate	95	0.001	186	2
5 mM caprylate	82	0.001	165	2
1 mM DL-lactate	103	0.35	213	1
5 mM DL-lactate	106	0.03	220	1
10 mM DL-lactate	102	0.62	189	1

In the 1-10 mM concentration range gluconate, glycerol, and inositol maximally extended lifespan by 14-21%, phophoenolpyruvate maximally extended lifespan by 12%, ribose maximally increased lifespan by 9%, xylose, galactose, or DL-lactate maximally increased lifespan by 6%, while glucuronolactone did not extend lifespan. 0.1 mM caprylate, an 8-carbon fatty acid, extended lifespan by 7%, while glyceraldehyde, fructose, propionate, and dihydroxyacetone did not extend lifespan in the 1-10 mM concentration range and even decreased lifespan by 13%, 14%, 18%, and 25%, respectively, at the 10 mM dose. Concentrations of glycerol over 100 mM have been previously shown to either not affect lifespan [1] or even decrease lifespan in *C. elegans* [2], while glycerol extended lifespan in rotifers [3]. It is of interest that we found galactose to slightly extend *C. elegans* lifespan, while it is known to induce premature senescence in fruit flies and rodents [4], while fructose decreased lifespan similarly to glucose. In addition to our findings here, lactate and gluconate have previously been shown to extend lifespan of Drosophila [5].

References

1. Deocaris CC, Takano S, Priyandoko D, Kaul Z, Yaguchi T, Kraft DC, Yamasaki K, Kaul SC, Wadhwa R: **Glycerol stimulates innate chaperoning, proteasomal and stress-resistance functions: implications for geronto-manipulation**. *Biogerontology* 2008, **9**(4):269-282.
2. Lee S-J, Murphy CT, Kenyon C: **Glucose Shortens the Life Span of C. elegans by Downregulating DAF-16/FOXO Activity and Aquaporin Gene Expression**. *Cell Metabolism* 2009, **10**(5):379-391.
3. Snell TW, Johnston RK: **Glycerol extends lifespan of Brachionus manjavacas (Rotifera) and protects against stressors**. *Exp Gerontol* 2014, **57C**:47-56.
4. Cui X, Wang L, Zuo P, Han Z, Fang Z, Li W, Liu J: **D-galactose-caused life shortening in Drosophila melanogaster and Musca domestica is associated with oxidative stress**. *Biogerontology* 2004, **5**(5):317-325.
5. Massie HR, Williams TR: **Increased longevity of Drosophila melanogaster with lactic and gluconic acids**. *Exp Gerontol* 1979, **14**(3):109-115.
