# Supplementary material for: Mechanisms of amino acid-mediated lifespan extension in Caenorhabditis elegans
Source: BMC Genet. 2015 Feb 3;16(1):8. doi: 10.1186/s12863-015-0167-2 (PMC4328591; doi:10.1186/s12863-015-0167-2)
Supplement: Additional file 6: Table S3. — The effects of nitrogen containing metabolites on C. elegans lifespan. [file 12863_2015_167_MOESM6_ESM.pdf]

Table S3. The effect of nitrogen containing metabolites on *C. elegans* lifespan

treatment	% of control mean lifespan	p-value	# of worms	replicates
0.125 g/L peptone	101	0.176	231	2
0.625 g/L peptone	90	<0.001	232	2
1.25 g/L peptone	77	<0.001	226	2
1 mM ammonium chloride	99	0.78	185	2
5 mM ammonium chloride	98	0.727	205	2
10 mM ammonium chloride	104	0.0789	238	2
1 mM creatine	96	0.001	267	2
5 mM creatine	80	<0.001	170	2
10 mm creatine	82	<0.001	118	2
0.01 mM carnosine	101	0.833	149	2
0.1 mM carnosine	114	<0.001	187	2
1 mM carnosine	111	0.0163	186	2
1 mM beta-alanine	113	<0.001	130	1
5 mM beta-alanine	105	0.871	228	1
0.1 mM ornithine	103	0.261	226	2
1 mM ornithine	108	<0.001	218	2
10 mM ornithine	90	<0.001	206	2
0.1 mM agmatine	101	0.627	223	2
1 mM agmatine	116	<0.001	212	2
10 mM agmatine	80	<0.001	220	2
0.1 mM putrescine	101	0.754	205	2
1 mm putrescine	110	<0.001	250	2
10 mM putrescine	87	<0.001	239	2
1 mM taurine	111	<0.001	261	2
5 mM taurine	106	0.0113	273	2
10 mM taurine	96	0.0237	231	2
0.1 mM theanine	114	0.007	130	1
1mM theanine	108	<0.001	225	1
5mM theanine	110	<0.001	172	1
10mM theanine	85	<0.001	183	1

0.1 mM histamine	103	0.719	161	2
1 mM histamine	93	0.00567	170	2
10 mM histamine	93	0.00437	191	2
1 mM betaine	109	0.009	132	1
0.1 mM urocanic acid	105	0.175	176	2
1 mM urocanic acid	97	0.213	146	2
10 mM urocanic acid	100	0.583	152	2
1 mM homocysteine	113	<0.001	140	1
5 mM homocysteine	107	0.0259	135	1
1 mM picolinic acid	107	0.126	148	1
1 mM quinolinic acid	74	<0.001	125	1

Creatine supplementation decreased lifespan up to 20% at these concentrations. The supplementation of the dipeptide antioxidant carnosine (beta-alanyl-L-histidine) or its precursor beta-alanine extended lifespan by 11-13% at a 1 mM dose. Betaine (trimethylglycine), an important cofactor for methylation events, extended lifespan by 9% at the 1 mM dose, while homocysteine, another intermediate in the methionine cycle extended lifespan by 13% at this concentration. The arginine breakdown products ornithine, agmatine and putrescine were able to maximally extend lifespan by 8-15% at the 1 mM dose, which is not surprising as they are precursors to the polyamine spermine, which has been shown to increase lifespan by promoting autophagy [1]. Taurine, a cytoprotective sulfonic acid and a derivative of cysteine, which can protect worms from ER stress through increased heat shock expression [2], extended lifespan by 11% at the 1 mM dose as well. We also confirmed data from others [3] that the amino acid theanine from 0.1 to 5 mM concentrations extended lifespan.

1. Eisenberg T, Knauer H, Schauer A, Buttner S, Ruckenstuhl C, Carmona-Gutierrez D, Ring J, Schroeder S, Magnes C, Antonacci L *et al*: **Induction of autophagy by spermidine promotes longevity**. *Nat Cell Biol* 2009, **11**(11):1305-1314.
2. Kim HM, Do CH, Lee DH: **Taurine reduces ER stress in C. elegans**. *J Biomed Sci* 2010, **17 Suppl 1**:S26.
3. Zarse K, Jabin S, Ristow M: **L-Theanine extends lifespan of adult Caenorhabditis elegans**. *Eur J Nutr* 2012, **51**(6):765-768.
