# Supplementary material for: Mechanisms of amino acid-mediated lifespan extension in Caenorhabditis elegans
Source: BMC Genet. 2015 Feb 3;16(1):8. doi: 10.1186/s12863-015-0167-2 (PMC4328591; doi:10.1186/s12863-015-0167-2)
Supplement: Additional file 8: Table S4. — The effects of amino acids on amyloid-beta-induced muscle paralysis. [file 12863_2015_167_MOESM8_ESM.pdf]

Table S4. The effect of amino acids on amyloid-beta-induced muscle paralysis

treatment	% of mean control time to paralysis	p-value	# of worms	replicates
10 mM serine	108.05	0.073	172	3
1 mM tryptophan	104.24	0.207	185	3
5 mM histidine	106.20	0.05	97	2
5 mM proline	99.14	0.896	85	1
1 mM methionine	98.99	0.739	163	3
30 mM methionine	81.02	0.001	88	1
