# Supplementary material for: Mechanisms of amino acid-mediated lifespan extension in Caenorhabditis elegans
Source: BMC Genet. 2015 Feb 3;16(1):8. doi: 10.1186/s12863-015-0167-2 (PMC4328591; doi:10.1186/s12863-015-0167-2)
Supplement: Additional file 10: Table S5. — The effects of amino acids or alpha-ketoglutarate on lifespan in human TDP-43 transgenic C. elegans. [file 12863_2015_167_MOESM10_ESM.pdf]

Table S5. The effect of amino acids or alpha-ketoglutarate on lifespan in human TDP-43 transgenic *C. elegans*

treatment	% of mean control lifespan	p-value	# of worms	replicates
10 mM serine	98	0.373	50	2
1 mM tryptophan	98	0.287	50	2
5 mM histidine	86	<0.001	47	2
5 mM proline	97	0.484	50	2
5 mM methionine	93	0.113	46	2
25 mM alpha-ketoglutarate	110	0.008	100	2
