# Supplementary material for: Mechanisms of amino acid-mediated lifespan extension in Caenorhabditis elegans
Source: BMC Genet. 2015 Feb 3;16(1):8. doi: 10.1186/s12863-015-0167-2 (PMC4328591; doi:10.1186/s12863-015-0167-2)
Supplement: Additional file 11: Table S6. — The effects of amino acids on C. elegans lifespan in the presence of 50 mM glucose. [file 12863_2015_167_MOESM11_ESM.pdf]

Table S6. The effect of amino acids on *C. elegans* lifespan in the presence of 50 mM glucose

treatment	% of mean lifespan in the presence of 50 mM glucose	p-value	# of worms	replicates
10 mM serine	114	<0.001	365	2
5 mM histidine	97	0.2910	334	2
5 mM glutamine	92	<0.001	120	2
5 mM proline	101	0.546	205	2
1 mM tyrosine	96	0.0870	172	2
1 mM tryptophan	110	<0.001	143	2
10 mM tryptophan	102	0.7340	112	2
